# Contrasting somatic mutation patterns in aging human neurons and oligodendrocytes

**DOI:** 10.1016/j.cell.2024.02.025

**Published:** 2024-03-18

**Authors:** Javier Ganz, Lovelace J. Luquette, Sara Bizzotto, Michael B. Miller, Zinan Zhou, Craig L. Bohrson, Hu Jin, Antuan V. Tran, Vinayak V. Viswanadham, Gannon McDonough, Katherine Brown, Yasmine Chahine, Brian Chhouk, Alon Galor, Peter J. Park, Christopher A. Walsh

**Affiliations:** 1 Division of Genetics and Genomics, Manton Center for Orphan Disease Research, Department of Pediatrics, and Howard Hughes Medical Institute, Boston Children’s Hospital, Boston, MA 02115, USA; 2 Departments of Pediatrics and Neurology, Harvard Medical School, Boston, MA 02115, USA; 3 Broad Institute of MIT and Harvard, Cambridge, MA 02142, USA; 4 Department of Biomedical Informatics, Harvard Medical School, Boston, MA 02115, USA; 5 Sorbonne Université, Institut du Cerveau (Paris Brain Institute) ICM, Inserm, CNRS, Hôpital de la Pitié Salpêtrière, 75013 Paris, France; 6 Department of Pathology, Brigham and Women’s Hospital, Harvard Medical School, Boston, MA 02115, USA; 7 Division of Genetics, Brigham and Women’s Hospital, Boston, MA 02115, USA; 8 These authors contributed equally; 9 Present address: Merck Research Laboratories, Cambridge, MA 02142, USA; 10 Lead contact

## Abstract

Characterizing somatic mutations in the brain is important for disentangling the complex mechanisms of aging, yet little is known about mutational patterns in different brain cell types. Here, we performed whole-genome sequencing (WGS) of 86 single oligodendrocytes, 20 mixed glia, and 56 single neurons from neurotypical individuals spanning 0.4–104 years of age and identified >92,000 somatic single-nucleotide variants (sSNVs) and small insertions/deletions (indels). Although both cell types accumulate somatic mutations linearly with age, oligodendrocytes accumulated sSNVs 81% faster than neurons and indels 28% slower than neurons. Correlation of mutations with single-nucleus RNA profiles and chromatin accessibility from the same brains revealed that oligodendrocyte mutations are enriched in inactive genomic regions and are distributed across the genome similarly to mutations in brain cancers. In contrast, neuronal mutations are enriched in open, transcriptionally active chromatin. These stark differences suggest an assortment of active mutagenic processes in oligodendrocytes and neurons.

## In brief

By employing single-cell whole-genome sequencing and integrating single-nucleus RNA-seq and single-nucleus ATAC-seq from the same individuals, this study uncovers distinct aging-related patterns of somatic mutation in human oligodendrocytes and neurons, which contribute to a deeper understanding of the mechanisms involved in human brain aging.

## Graphical Abstract

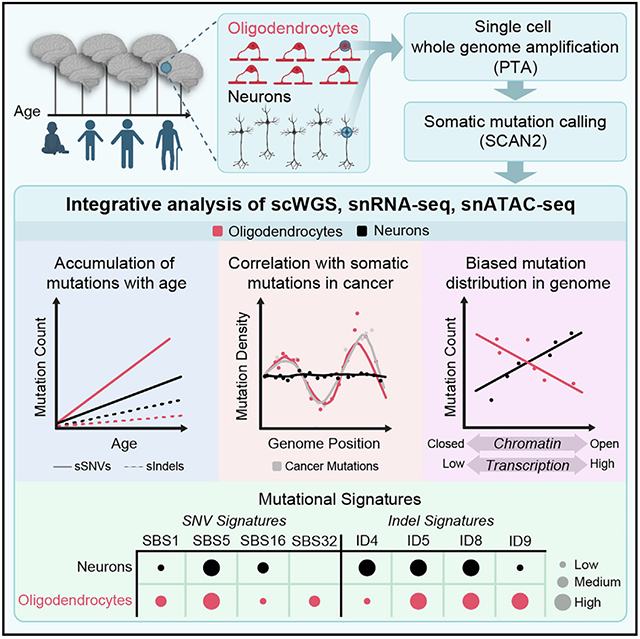

## INTRODUCTION

Somatic mutations accumulate in every tissue of the human body throughout life, via mechanisms that depend on intrinsic tissue physiology and exogenous agents.^1-8^ Because human tissues comprise diverse cell types with unique properties, quantifying cell-type-specific rates and mechanisms of somatic mutation is fundamental to understanding aging and disease initiation at the tissue level. Although previous studies have addressed somatic mutations in aging human neurons,^4,9-11^ mutations in glial cells, which represent more than half of the cellular content of the brain and play primary roles in several brain disorders, have not yet been examined.

Oligodendrocytes (OLs) are the main cell type of the white matter (WM),^12^ whose degeneration is considered to be a hall-mark of normal brain aging^13-15^ and neurodegenerative disorders.^16-18^ A recent multi-omic study in mice reported accelerated glial aging in cortical regions, implicating WM as vulnerable foci during aging.^19^ Abnormalities in OLs have been reported in age-related^20-22^ and psychiatric disorders,^23,24^ glial-derived brain tumors,^25,26^ and immune-related multiple sclerosis.^27,28^ OL generation in humans begins during the first trimester of gestation, peaks at birth and during the first years of life, and continues into adulthood, though at reduced rates.^29-32^ Unlike neurons, which mostly arise before birth, OLs are replenished throughout postnatal life by resident OL-precursor cells (OPCs),^29,33^ with the rate of replenishment diminishing with age.^34,35^ Dysregulation of proliferation and differentiation in the OL lineage is involved in brain cancer, and OPCs are recognized as the cell of origin in some gliomas.^25,26,36,37^ Thus, in contrast to neurons, OLs may be subject to mutational processes related to DNA replication and can potentially undergo positive selection relevant for cancer insurgence.^38^ Consistent with this notion, recent findings have shown enriched clonal oncogenic mutations within the WM of non-diseased human brains.^39^

**Figure 1.**
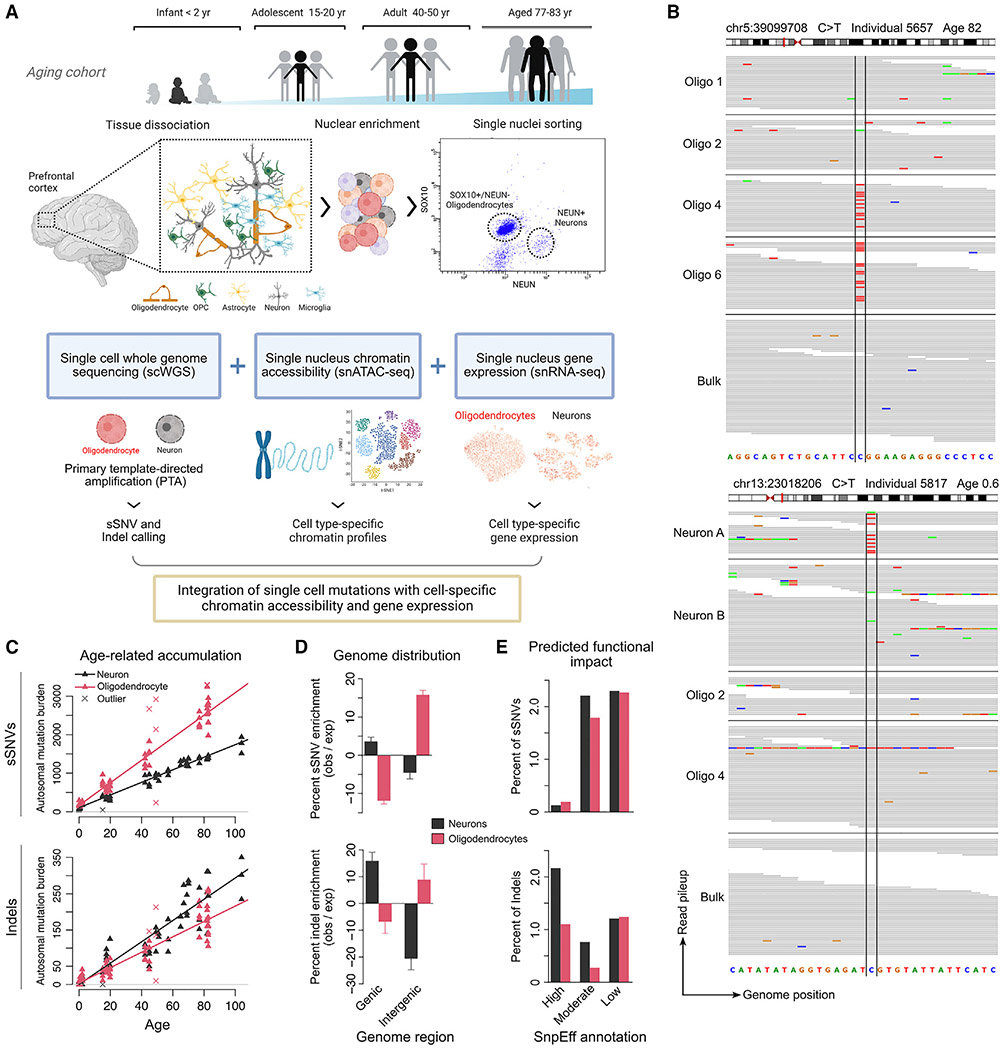
Somatic mutations in neurons and oligodendrocytes accumulate at different rates and in different genomic regions (A) Experimental strategy. Oligodendrocytes (OL; n = 66 PTA, n = 20 MDA) and neurons (n = 56 PTA) were obtained from the brains of 20 neurotypical individuals (0–104 years of age) through FANS using NEUN (neurons) and SOX10 (OL) antibodies. Single genomes were amplified using PTA or MDA and non-clonal somatic SNVs (sSNVs) and indels were called using SCAN2. Mutation distributions were compared with snATAC-seq and snRNA-seq data obtained from a subset of the 20 individuals. (B) Integrated Genomics Viewer screenshots of two sSNVs identified by SCAN2. Top, an sSNV shared by two oligodendrocytes; bottom, a private sSNV in a neuron. (C) Extrapolated genome-wide sSNV and indel burdens for OLs and neurons as a function of age. SCAN2 estimates mutation burdens for each single cell individually by adjusting for sensitivity. Trend lines are mixed-effects linear regression models; outlier single cells with abnormally high or low mutation burdens, indicated by crosses, were excluded from the linear regressions (see STAR Methods). (D) Distribution of OL and neuronal sSNVs and indels in annotated gene regions. Enrichment/depletion levels are calculated by comparison with a null distribution obtained by randomly shuffling mutations across the genome followed by correction for somatic mutation detection sensitivity; error bars represent bootstrapped 95% CIs (see STAR Methods). Percentages give the observed mutation count divided by the expected mutation count from the null distribution in each region. (E) Percent of somatic mutations in the total mutation catalog with HIGH, MODERATE, and LOW impact on genes, as determined by SnpEff. See also Figures S1, S2, S3, and S4 and Tables S1, S2, and S3.

In this study, we assessed genome-wide rates and patterns of aging-related somatic mutations in OLs compared with neurons isolated from the same individuals using single-cell whole-genome sequencing (scWGS). In addition, we generated single-nucleus assay for transposase-accessible chromatin with sequencing (snATAC-seq) data from these brains and integrated new as well as published^8^ single-nucleus RNA sequencing (snRNA-seq) data from individuals in the same cohort (Figure 1A). With joint analysis of these data, we inferred OL- and neuron-specific rates and patterns of somatic mutation accumulation, with DNA replication and transcription playing significant roles in OL and neuronal mutagenesis, respectively. We also captured features of mutational processes in the differentiated OLs as well as in precursor OPCs. The substantial differences in somatic mutation rate and localization between these two adjacent and interacting cell types are likely to be vital for elucidating cell-type-specific contributions to age-related diseases.

## RESULTS

### OLs accumulate somatic mutations at different rates than neurons

OLs were isolated by antibody staining of nuclei prepared from post-mortem cortical brain tissue, selecting SOX10-positive and NEUN-negative nuclei by fluorescence-activated nuclear sorting (FANS). snRNA-seq performed on the sorted populations confirmed >99% purity for both mature OLs and neurons sorted by SOX10 and NEUN positivity, respectively. Further assessment by droplet digital PCR of SOX10-positive, NEUN-negative nuclei indicated at least 89% purity for mature OLs, while 6.9% expressed CSPG4 and/or PDGFRA (possible OPCs), 1.3% were negative for OPC and OL markers, and 2.6% were positive for both OLs and OPC markers, likely indicating developmental transitions between these cells (Figure S1; STAR Methods).

Overall, 86 OLs were obtained from the prefrontal cortex (PFC) of 13 neurologically normal individuals spanning 0.4–83 years of age (Tables S1 and S2): 66 single-OL genomes were amplified by primary template-directed amplification (PTA), a recent technique that substantially improves amplification quality,^10,40^ and 20 were amplified by multiple-displacement amplification (MDA) before PTA became available. An additional set of 20 GFAP-positive, NEUN-negative single cells, which represent a mixed population that are predominantly OPCs (Figure S1B), were also amplified by MDA. Due to the higher rate of technical artifacts caused by MDA,^10^ we focused on PTA-amplified samples except where indicated. For OL vs. neuron comparison, we used 56 PTA-amplified neurons (52 previously generated^10^ and 4 new) from 19 individuals, 12 of which overlap our OL cohort.

Following scWGS, somatic single-nucleotide variants (sSNVs) and small (1–30 base pair [bp]) insertions/deletions (indels) were identified genome-wide using SCAN2^10^ (Figure 1B; STAR Methods), an algorithm we recently developed to call somatic mutations in PTA-amplified single cells with high specificity and to accurately extrapolate the total mutation burden per cell from the observed number of mutations by adjusting for sensitivity (Table S3). To focus on somatic mutations acquired during aging rather than development, high allele frequency clonal sSNVs and indels were excluded by removing somatic calls supported by one or more reads in matched 30–45× bulk DNA sequencing. One component of SCAN2 mutation calling involves analysis of mutational signatures. These mutation calls were used only for enrichment analyses (with appropriate adjustment) but not for any analysis of mutational spectra or total burden. Finally, unless otherwise noted, recurrent somatic calls were either removed if they appeared in multiple individuals (suggesting artifactual origin) or downsampled to a single representative occurrence if limited to one individual (suggesting shared line-age, STAR Methods). PTA and SCAN2 enabled broad coverage of the genome and accurate mutation calling (48% sensitivity and 6%–8% false positive rate for sSNVs; 41% sensitivity and 3%–7% false positive rate for indels; Figure S2; STAR Methods). In addition to our estimates of mutation detection accuracy, two recent studies employing different duplex sequencing approaches to study somatic mutations in human neurons^9,41^ confirmed our estimates of neuronal mutation rates, per-cell mutation burdens, and mutational signatures (Figure S3), providing orthogonal confirmation of our approach.

Compared with neurons, scWGS of OLs revealed higher yearly rates of sSNV accumulation but lower rates of indel accumulation. As is the case with neurons and many other cell types,^3,4,9-11,42^ the increase in OL sSNV burden was remarkably linear with respect to age, with a rate of 29 sSNVs/year (95% confidence interval [CI]: 27.6–30.9), which is significantly greater than the neuronal rate of 16 sSNVs/year (CI: 15.2–17.5, Figure 1C; for the difference, p = 1.54 × 10^−26^, t test for coefficients in a linear mixed model [LMM], see STAR Methods). At birth, OLs contained 54% more sSNVs per genome compared with neurons (intercept: 165 vs. 107), though this difference was not significant (p = 0.24, LMM t test). Similar rates were observed for MDA-amplified OLs (30 sSNVs/year) and mixed glia (30 sSNVs/year) (Figure S4A; STAR Methods). Unlike sSNVs, indels accumulated more slowly in OLs than in neurons (2.1 [CI: 1.90–2.34] versus 2.9 [CI: 2.47–3.40] indels/year, respectively, p = 0.0006, LMM t test, Figure 1C). Indel burdens at birth were comparable between cell types. Deletions were more prevalent than insertions in both cell types, consistent with previous reports^10,43^ (Figure S4B); however, OL indels were mostly single-bp deletions, while neurons carried greater numbers of 2–4 bp deletions and 1 bp insertions (Figure S4C), likely representing distinct mechanisms of indel generation.

OL and neuronal mutations showed opposite biases for genic regions, suggesting different mechanisms of mutagenesis and different consequences for gene integrity. After correcting for local mutation detection sensitivity (Figure S4D; STAR Methods), OL sSNVs were significantly enriched in intergenic regions, with 15.8% more mutations than expected (Figure 1D; p < 10^−4^, all p values for enrichment analyses based on permutation tests, see STAR Methods) and depleted in genic regions, with 11.9% fewer than expected (p < 10^−4^). This pattern was replicated in MDA-amplified OLs from elderly individuals (Figure S4E; STAR Methods). In contrast, neuronal sSNVs were overrepresented in genes (3.6%, p < 10^−4^) and depleted in intergenic regions (4.6%, p < 10^−4^). Indels mirrored these patterns but with greater effect sizes in neurons: OL indels were enriched by 8.9% (p = 0.003) in intergenic regions and depleted by 6.8% (p = 0.001) in genes. Neuronal indels were instead depleted by 20.6% (p < 10^−4^) in intergenic regions and enriched by 15.9% (p < 10^−4^) in genes, as previously reported.^10^ In general, a larger fraction of neuronal mutations were predicted by SnpEff^44^ to functionally impact genes (Figure 1E). Strikingly, the rate of indels with the most severe gene-altering effects was ~2-fold higher in neurons than in OLs. Due to the small number of mutations in genes and large effect of multiple hypothesis testing correction, no significant mutation enrichment or depletion was detected for any individual gene (Figure S4F).

### OL mutagenesis is marked by signatures of cell proliferation and aging

**Figure 2.**
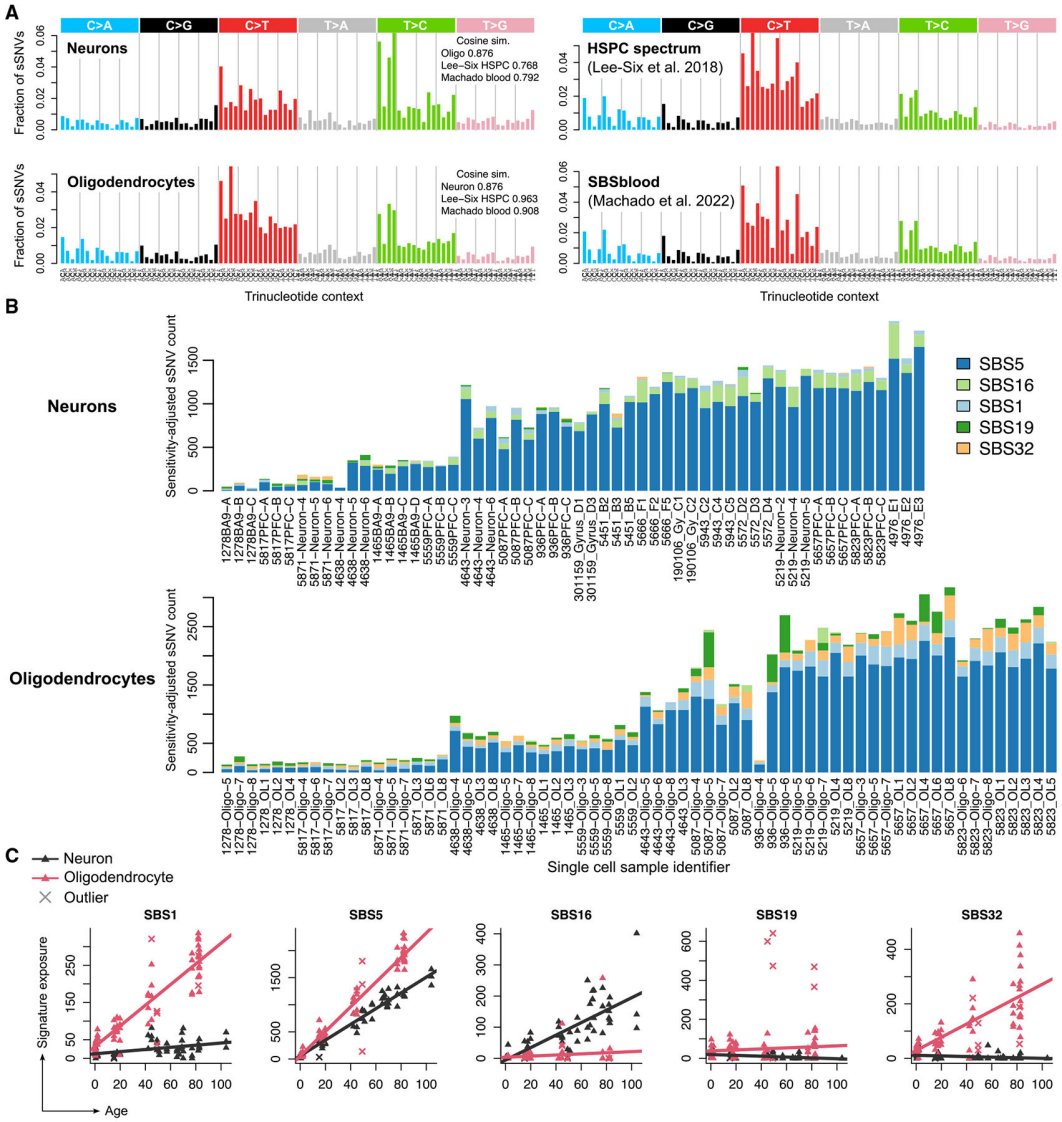
The composition of somatic SNVs, reflected in exposure to COSMIC mutational signatures, also differs between neurons and oligodendrocytes (A) SBS mutational spectra of neuronal and oligodendrocyte sSNVs identified in this study (left column); the spectrum of hematopoietic stem and progenitor cells (HSPCs) identified in Lee-Six et al.,^45^ and a signature derived from an analysis of human lymphocytes (Machado et al.^46^). Cosine similarities are shown for each pair of spectra. (B) The number of somatic mutations, after extrapolation to genome-wide burdens, attributed to each COSMIC SBS signature by SigProfilerExtractor for each PTA single OL and neuron. Subjects are ordered from young (left) to elderly (right). (C) Same signature exposure values in (B) plotted against age. Each point represents one single cell. Crosses indicate the outlier cells, in terms of total mutation burden, as identified in Figure 1C. Trend lines are linear regression models from which outliers were excluded (see STAR Methods). See also Figure S4.

Analysis of mutational spectra and signatures indicated shared and cell-type-specific mutational mechanisms in OLs and neurons. The spectrum of OL sSNVs matched the spectrum of highly proliferative hematopoietic stem and progenitor cells (HSPCs, cosine similarity 0.96)^9,42,45,46^ more closely than the spectrum of neuronal sSNVs did (cosine similarity 0.77, Figure 2A). The OL spectrum was less similar to neurons (cosine similarity 0.89) than to HSPCs, suggesting shared somatic mutagenic processes between OLs and HSPCs.

To explore mutagenic mechanisms, we quantified exposure to single-base substitution (SBS) mutational signatures from the COSMIC catalog (v3.3)^47^ using SigProfilerExtractor.^48^ We identified five active COSMIC SBS signatures in either OLs or neurons (Figure 2B; STAR Methods). Signature SBS5, a clock-like signature that accumulates independently of cell division, was the most prevalent signature in both cell types and it accumulated at a significantly higher rate in OLs compared with neurons (22.7 versus 14.5 sSNVs/year, p < 10^−16^, Figures 2B and 2C, LMM t test). Signatures SBS1 and SBS32 were strongly associated with age in OLs (p < 10^−16^ for SBS1, p = 1.3 × 10^−12^ for SBS32, LMM t test) but were nearly absent in neurons (p = 0.001 and p = 0.03, respectively, LMM t test). SBS1 is a clock-like signature associated with cell division^49^ and accumulated at rates of 2.77 and 0.29 sSNVs/year in OLs and neurons, respectively (Figures 2B and 2C). Because mutations in mature OLs represent a mixture of mutations gained at the OPC stage and the post-mitotic OL stage, it is possible that SBS1 mutations were generated primarily during the OPC stage as a result of OPC mitosis. SBS32 is a C>T signature that was recently found to differentiate the mutational spectrum of HSPCs from that of the colon, liver, and intestine.^42^ Only SBS16, a signature associated with transcription, accumulated at a higher rate in neurons (2.0 sSNVs/year) than in OLs (0.18 sSNVs/year), consistent with the enrichment of neuronal mutations in transcribed genomic regions and in line with previous reports.^4,10^ SBS19 is a rarely observed signature of unknown etiology, though it has been observed in small numbers of low-grade gliomas and pilocytic astrocytomas. Recently, SBS19 was estimated to contribute 2–4 mutations per year in HSPCs as a result of lesion bypass mechanisms interacting with persistent DNA damage.^50^ In our data, it featured primarily in outlier OLs and did not significantly correlate with age (p = 0.10). Two possible explanations for our observed SBS19 levels are (1) technical artifacts—though this does not explain why SBS19 was not observed at appreciable levels in any of our 56 neurons—or (2) an atypical mutational process. Age-related accumulation of SBS signatures was similar between PTA OLs, MDA OLs, and MDA mixed glia, with the notable exception of SBS1, which was elevated in MDA mixed glia (Figure S4G), consistent with the higher proportion of OPCs (58%)—a mitotic cell type—in this population.

**Figure 3.**
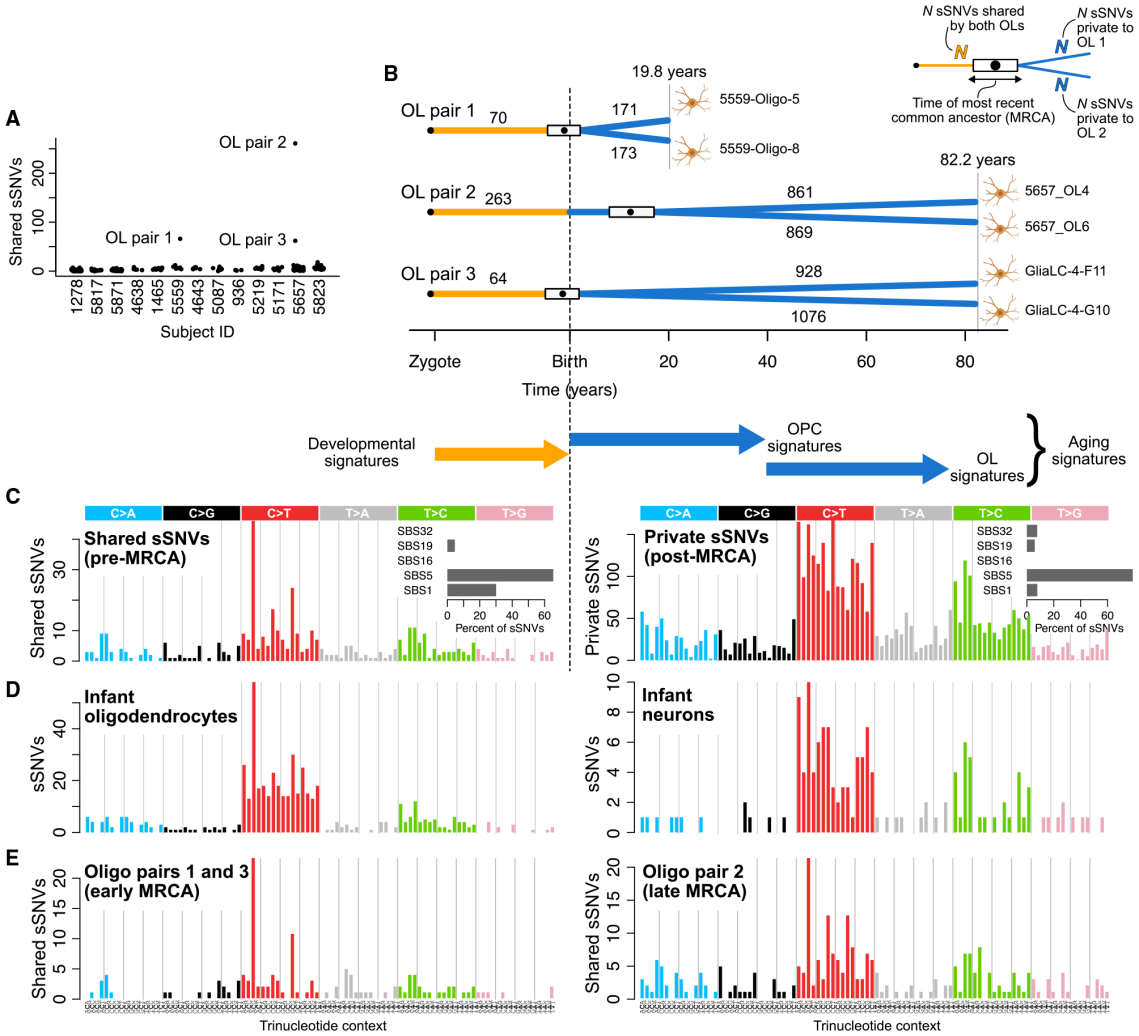
Shared somatic sSNVs of oligodendrocyte pairs reveal mutational characteristics of oligodendrocyte precursor cells (OPCs) (A) Number of sSNVs shared between every pair of OLs for each individual in this study. (B) Schematic of three pairs of related OLs and estimates of the time of division for each pair’s most recent common ancestor (MRCA), with the box providing a range (not a confidence interval) derived from the 95% confidence intervals on the OL aging accumulation model and the point providing a single best estimate (see STAR Methods). (C) The SBS mutational spectrum and contributions of COSMIC signatures (insets) for sSNVs acquired before division of the MRCA (shared sSNVs) and sSNVs acquired after division of the MRCA (private sSNVs) shows greater contribution of SBS1 at earlier stages. (D) SBS mutational spectra for high-confidence mutations from infant (0–2 years of age) PTA OLs and neurons (STAR Methods). (E) SBS mutational spectra for shared sSNVs from OL pairs with early (pairs 1 and 3) and late (pair 2) MRCAs.

Three pairs of closely related OLs, which likely trace their ancestry to common OPCs, allowed us to investigate the differences between early- and late-life mutational processes. Despite filtering high allele frequency clonal sSNVs, three OL pairs from two individuals (subjects UMB5559 and UMB5657, 19.8 and 82 years, respectively) shared unusually high levels of sSNVs (70, 263, and 64 sSNVs, respectively, Figure 3A), indicating common ancestry. We estimated the age at which the most recent common ancestor (MRCA) OPC divided for each pair by comparing the number of shared sSNVs, corrected for detection sensitivity, to the OL aging trend line (see STAR Methods). This placed the MRCAs of pairs 1 and 3 near birth and at ~12 years for pair 2 (Figure 3B). In both subjects, the shared sSNVs were mostly C>T transitions at CpG sites, with a 30% contribution from the cell-division-related signature SBS1 (Figure 3C). Each pair of OLs also contained similar numbers of private sSNVs, consistent with equal lifetimes for each of the cognate OLs after the division of their MRCA. The mutational spectrum of private sSNVs was similar to the OL spectrum (Figure 2A) and was primarily explained by SBS5 (79%), followed by SBS1, SBS32, and SBS19 (7.7%, 7.6%, and 5.8%, respectively, Figure 3C).

Further comparisons of mutation spectra provided additional insight into MRCA timing and cell-type-specific mutational processes. First, we confirmed the timing of MRCAs by comparing the spectra of pre-MRCA mutations to those of neurons and OLs from infant subjects (aged 0–2 years old), which should contain mostly developmental signatures (Figure 3C). Indeed, the spectrum of shared sSNVs resembled the OL infant spectrum (cosine similarity = 0.89) more than the neuron infant spectrum (cosine similarity = 0.76). Crucially, the neuronal sSNV spectrum from the same infant subjects contained far fewer SBS1-like C>Ts at CpGs, implying that increased SBS1 is an indicator of OL-specific lineages and does not reflect early clonal sSNVs that may have evaded our filters. Next, because the MRCA of pair 2 occurred later in life than pairs 1 and 3, its shared sSNV spectrum should reflect greater exposure to OPC aging signatures while pairs 1 and 3 should be dominated by OPC developmental signatures. Comparison of the spectra revealed a noticeably larger exposure to SBS1-like mutations (C>Ts at NpCpG dinucleotides) in pairs 1 and 3, consistent with greater cell proliferation in the perinatal period^29^ (Figure 3E). Pair 2’s spectrum was more similar to the OL aging signature (cosine similarity 0.73 vs. 0.87), indicating a shift in OPC mutational processes during the first decade of life and suggesting that our OL mutation catalog contains a considerable number of mutations acquired at the OPC stage, though quantifying this is difficult without direct sequencing of OPCs. In summary, the mutational spectra provide further evidence that the MRCA lineages split during a burst of OPC proliferation and OL generation that occurs in the young human brain (0–10 years of age).^29^ The relationships of these three pairs of cells suggest that shared mutations mark a permanent forensic lineage tree, while non-shared mutations represent a linear timer of when any two cells separate from a common progenitor.

**Figure 4.**
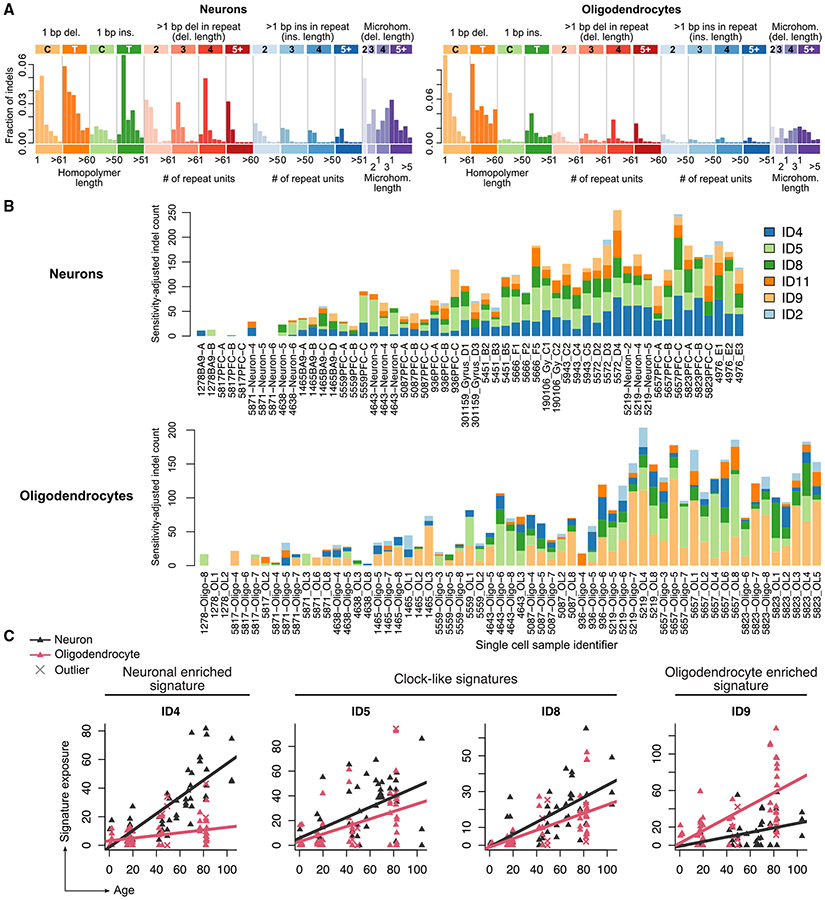
Insertion and deletion COSMIC signatures in human oligodendrocytes and neurons suggest differing mutagenic mechanisms (A) Spectra of somatic indels from human OLs and neurons using the 83-dimensional indel classification scheme from COSMIC. (B) Contribution of COSMIC indel signatures to each single OL and neuron. One bar represents one single cell; cells are ordered according to age, with the youngest individuals on the left and eldest individuals on the right. (C) Same as (B), but signature exposure is plotted against age for each single cell; each point represents one cell and crosses represent total mutational burden outliers. Trend lines are linear regression models from which outliers are excluded (see STAR Methods). ID5 and ID8 are annotated as clock-like signatures in COSMIC.

Indel signatures revealed shared and cell-type-specific mutational processes, further distinguishing OLs from neurons (Figures 4A-4C). ID4, a signature representing ≥2 bp deletions and associated with transcriptional mutagenesis,^51^ was most strongly correlated with age in neurons, as previously reported,^9,10^ but was almost completely absent in OLs (0.09 indels/year, p = 0.0007, LMM t test; Figure 4C). ID5 and ID8, two clock-like indel signatures, were present in both cell types, with ID8 correlated more strongly with age in neurons than in OLs. The two remaining clock-like indel signatures, ID1 and ID2, were either not detected (ID1) or detected at low levels (ID2, 0.02 indels/year and 0.1 indels/year in neurons and OLs, respectively), but they are difficult to identify due to similarity with sequencing artifacts.^10^ ID9, which is characterized by 1 bp deletions, was the most prevalent signature in OLs and accumulated at a rate of 0.69 indels/year; in neurons, the accumulation was significantly lower at 0.25 indels/year (p = 0.004, LMM t test). Interestingly, this ID9 signature is also found in a large fraction of adult gliomas^52^ as well as in a considerable fraction of other brain tumors.^47^

### OL sSNVs are enriched in inactive genomic regions

**Figure 5.**
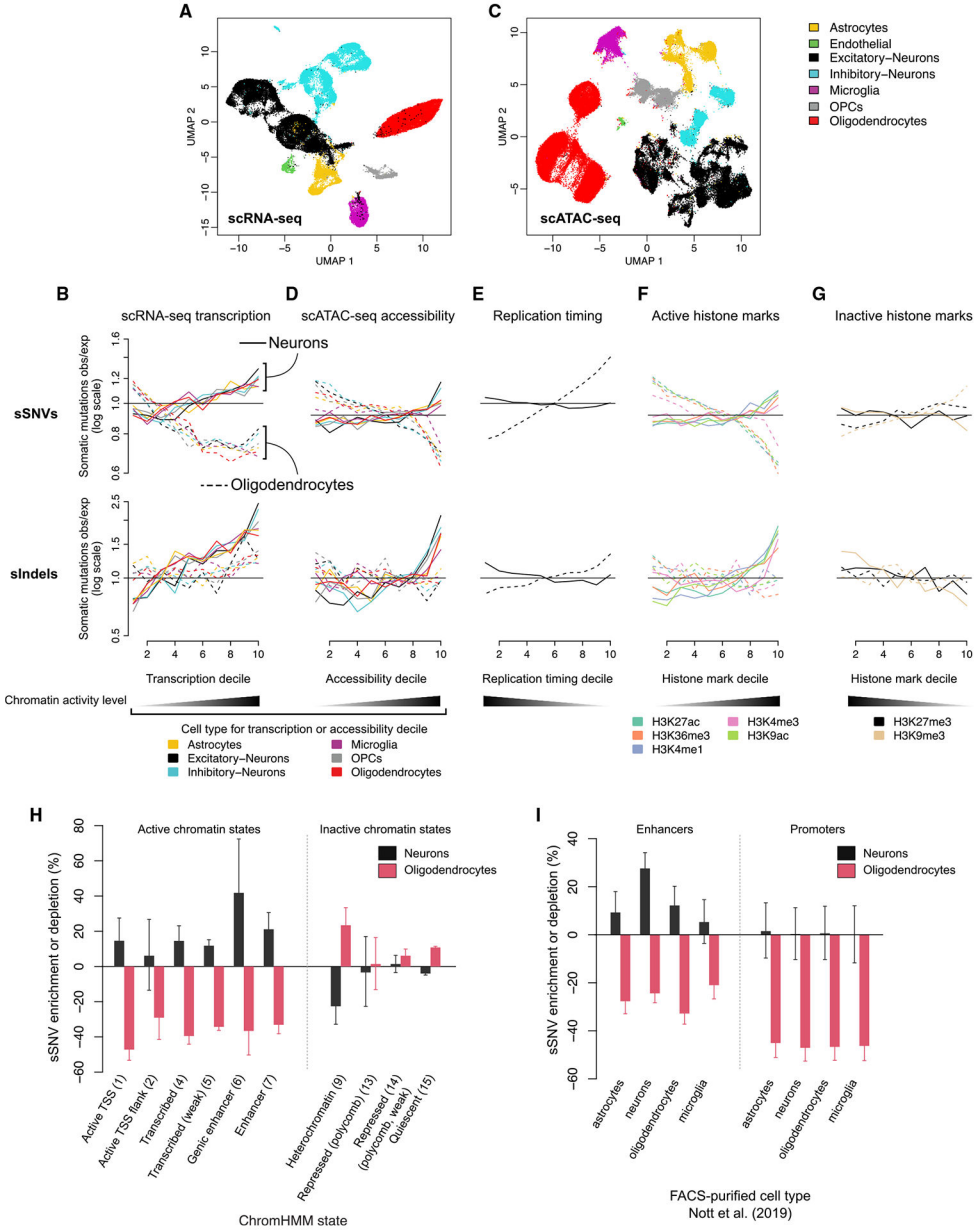
Oligodendrocyte somatic mutations are associated with inactive chromatin, while neuronal mutations associate with active chromatin (A) Uniform manifold approximation and projection (UMAP) plot of integrated snRNA-seq from three subjects (UMB1465, UMB4638, and UMB4643) with cell type annotations. (B) Enrichment analysis of somatic mutations vs. snRNA-seq transcription level. The genome is divided into 1 kb, non-overlapping windows, and each window is annotated with an average gene expression level per cell type; windows that are <20% covered by a gene are discarded. The remaining windows are classified into 10 deciles, with 1 representing the least transcribed and 10 representing the most transcribed. In each decile, the observed number of somatic SNVs and indels is compared with a null distribution of mutations obtained by randomly shuffling mutation positions followed by correction for somatic mutation detection sensitivity (see STAR Methods). Each line shows somatic mutation density vs. transcription level from one cell type identified in our snRNA-seq; solid lines indicate mutation density measured in PTA neurons and dashed lines indicate PTA oligodendrocytes. (C and D) Same as (A) and (B) for snATAC-seq from the brains of 10 subjects from this cohort. (E) Enrichment analysis of replication timing, as measured by ENCODE RepliSeq; lines represent average enrichment across 15 cell lines. (F and G) Enrichment analysis of 5 epigenetic marks related to gene activity (F) and two repressive epigenetic marks (G) measured in dorsolateral prefrontal cortex tissue (Roadmap Epigenomic Project, reference epigenome E073). (H and I) Enrichment analysis of functional genomic regions identified by ChromHMM in reference epigenome E073 (H) or active enhancers and promoters identified in Nott et al.^54^ for several brain cell types (I). Numbers in parentheses indicate the ChromHMM state number (H). Error bars represent bootstrapped 95% CIs (see STAR Methods). See also Figure S5.

Our earlier observation that OL mutations were depletedin genes—opposite to the pattern of neuronal mutations (Figure 1D)—suggested different determinants of mutagenesis in these two cell types. Comparison of somatic mutation density, after correction for location-specific mutation detection sensitivity (Figure S5A), to additional data types, including snRNA-seq, snATAC-seq, replication timing, and chromatin marks, revealed that OL mutations are enriched in chromatin that is either inaccessible, untranscribed, or which harbors repressive histone marks—which we refer to as inactive chromatin—in striking contrast to neuronal mutations. We first compared somatic mutation densities with gene expression levels from brain snRNA-seq data for three subjects in our cohort (UMB1465, UMB4638, and UMB4643; 40,083 PFC cells in total; Figure 5A; STAR Methods).^8^ OL sSNVs were depleted by 29%–33% in the top few deciles of expression measured in OLs (Figure 5B, p < 10^−4^; all p values in this section are from permutation tests) and similar depletion trends were observed for all other cell types. However, we note that power to detect cell-type-specific mutation enrichment signals is limited due to a high correlation between transcription levels (Pearson correlation range between pairs of cell types, 0.859–0.939). The negative association between transcription level and somatic mutation density in OLs was confirmed using bulk RNA-seq data from 54 diverse tissue types from the Genotype Tissue Expression Consortium (GTEx)^53^ (Figure S5B). Indels in OLs were not significantly enriched or depleted, possibly due to a lack of statistical power caused by the relatively low number of somatic indels in OLs (Figure 5B).

Next, brain snATAC-seq data representing ~82,000 cortical cells obtained from ten subjects in our cohort (see STAR Methods) revealed a strong depletion of OL sSNVs in open chromatin (Figures 5C and 5D). In the decile of the genome with the highest chromatin accessibility from OLs identified in snATAC-seq data, OL sSNVs were depleted by 30% (p < 10^−4^). Slightly weaker sSNV depletions were observed for the remaining cell types (mean 21% for the top decile of chromatin accessibility), with OPCs showing the second-strongest depletion signal (26%, p < 10^−4^). A weak but negative trend between OL indel density and chromatin accessibility was also observed (Figure 5D).

Data from the Encyclopedia of DNA Elements (ENCODE)^55^ and the Roadmap Epigenomics Project^56^ further confirmed enrichment of OL mutations in inactive chromatin. First, OL sSNVs were significantly enriched in late-replicating regions of the genome (which tend to be less transcriptionally active), as determined by RepliSeq data from the ENCODE project (mean 38% in the latest replicated decile, p < 10^−4^; Figures 5E and S5C). Comparison with histone marks from the Roadmap Epigenomics Project revealed negative associations between OL sSNVs and marks of open chromatin, transcription, and active regulatory elements (H3K27ac, H3K36me3, H3K4me1, H3K4me3, and H3K9ac) and positive associations with the repressive mark H3K9me3^57^ (Figures 5F and 5G). Chromatin state annotations from ChromHMM,^57^ which classify chromatin based on an ensemble of histone marks, further confirmed the pattern of OL mutation enrichment in inactive or inaccessible genomic regions, with OL sSNVs overrepresented in heterochromatin (state 9, 23% enrichment, p < 10^−4^) and quiescent regions (state 15, 11% enrichment, p < 10^−4^) and depleted in transcriptionally active states 1–7 (Figure 5H). The strongest depletion of OL sSNVs across all genomic covariates analyzed in this study was observed for active transcription start sites (ChromHMM state 1, 47.2% depletion). An orthogonal dataset of active promoters in neurons, OLs, microglia, and astrocytes from flow-sorted cell populations^54^ further confirmed the strong depletion of OL sSNVs in promoters (mean depletion 53.7%), and again there was no marked preference for the cell type from which the promoters were measured (Figure 5I).

The distribution of neuronal mutations differed from OLs across all the genomic covariates we tested: neuronal sSNV and indel rates increased with gene expression, chromatin accessibility and active histone modifications and decreased with inactive histone modifications (Figures 5A-5G). Unlike OLs, somatic mutations in neurons were more specifically associated with transcription levels measured in brain tissues (Figure S5B) and especially with single-cell transcriptomic and chromatin accessibility signals from neurons (Figures 5B and 5D). Neuronal mutations showed little association with replication timing (Figure 5E), which is unsurprising because most neuronal mutations are acquired in the post-mitotic state, and clonal somatic mutations were largely removed by our bulk filters.

**Figure 6.**
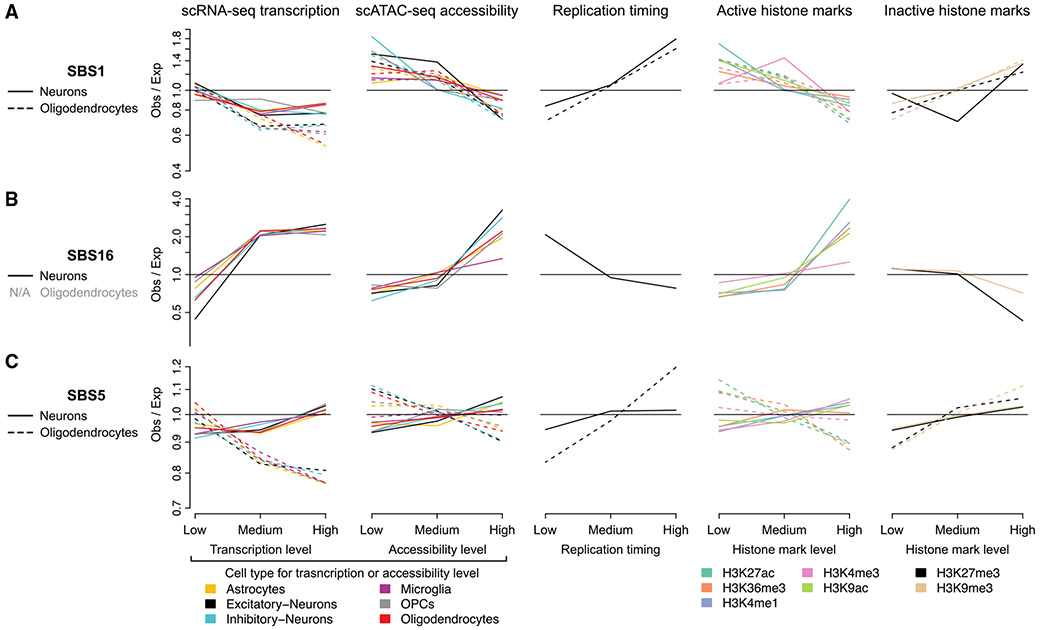
Distinct mutational signatures show cell-type-specific enrichment in active or inactive chromatin (A–C) Enrichment analysis of somatic mutations attributed to SBS1 (A), SBS16 (B), or SBS5 (C)—rather than total mutation density—vs. the decile-based genomic covariates presented in Figure 5. The genome was divided into three quantiles—rather than ten—to reduce noise in signature fitting caused by the smaller number of mutations attributed to each signature. OLs are not plotted for SBS16 due to near-complete lack of SBS16, leading to highly noisy measurements. See also Figure S6.

To further understand the action of mutational processes in the two cell types, we correlated SBS mutation signature exposures (rather than total mutation density) to the previously discussed genomic covariates (STAR Methods). To account for the smaller number of mutations assigned to individual signatures and to obtain sufficient mutations for signature fitting, the genome was binned into just three quantiles rather than ten. In OLs, SBS1 density generally followed the patterns of total mutation density, with positive associations with inactive chromatin and late replication timing (Figure 6A). The distribution of SBS1 in neurons mimicked that of OLs and was strongly positively associated with replication timing, suggesting that neuronal SBS1 may have accumulated during cell divisions in neurogenesis. SBS16 (a T>C signature associated with transcriptional activity) exposure in neurons was positively associated with active histone marks, gene expression, and chromatin accessibility levels from excitatory and inhibitory neurons (Figure 6B). Consistent with the known transcribed-strand bias of SBS16, neuronal T>C mutations exhibited significant transcribed-strand bias (p = 0.0005, Wilcoxon rank-sum test, Figure S6). Interestingly, despite neurons being post-mitotic, SBS16 density trended negatively with replication timing, likely reflecting higher gene density in early replicating regions.

Although SBS5 is the most prevalent signature in both OLs and neurons, it did not accumulate in the same genomic regions in these two cell types, particularly with respect to expression levels (Figure 6C). In OLs, patterns of SBS5 exposure showed little difference from the aggregate somatic mutation density, with negative associations with active epigenetic marks, gene expression, and open chromatin and positive associations with inactive marks and late-replicating regions. However, in neurons, unlike aggregate mutation density, SBS5 was only moderately associated with the covariates tested (enrichment or depletion < 8%), with the strongest associations deriving from snRNA-seq expression (Figure 6C). These observations suggest that either SBS5 is generated by cell-type-specific mechanisms or that SBS5 may not be a fully decomposed signature—in particular, it may be contaminated by the transcription-associated SBS16, consistent with the marginally significant association with expression levels in neurons—as previously suggested.^9^

### The OL mutation density profile resembles that of glial-derived tumors

**Figure 7.**
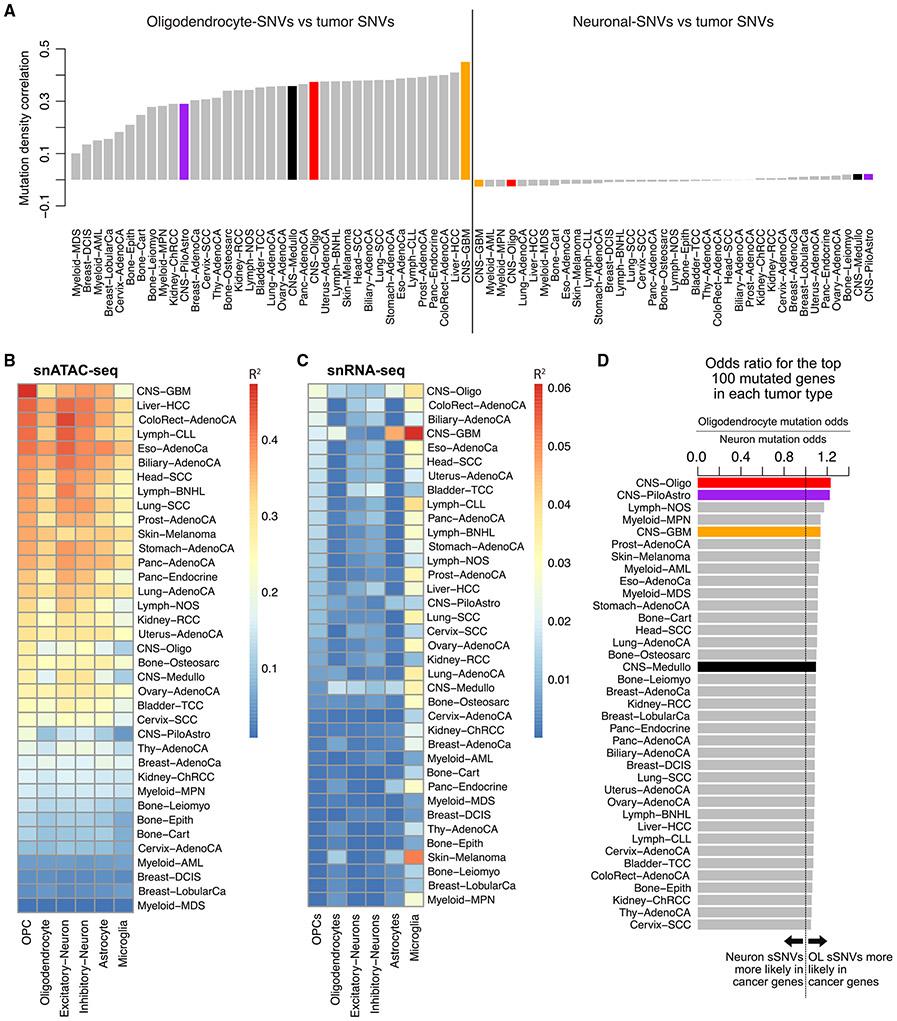
Patterns of oligodendrocyte sSNVs correlate with somatic mutation density in cancer (A) Correlation of OL and neuronal sSNV mutation density with cancer mutation density. For each cell type and cancer type, the genome was tiled with non-overlapping 1 MB bins and numbers of mutations per bin were tabulated. Somatic mutations from PTA neurons and PTA OLs were tabulated for the same regions and corrected for mutation detection sensitivity. CNS tumors are colored: CNS-Oligo, oligodendroglioma, red; CNS-PiloAstro, pilocytic astrocytoma, purple; CNS-GBM, glioblastoma multiforme, orange; CNS-Medullo, medulloblastoma, black. (B and C) Mutation density for each tumor type was fit using a linear regression to cell-type-specific single-cell chromatin accessibility signals from our snATAC-seq (B) and single-cell expression levels from our snRNA-seq (C) using the same 1 MB bins described in (A). For each tumor type and cell type, the fraction of variance in tumor mutation density explained ($R^{2}$) by each cell type is shown. (D) Comparison of OL and neuron somatic mutation rates in frequently mutated cancer genes. For each tumor type in PCAWG (y axis), the 100 most-frequently mutated genes were determined. For each tumor-specific set of 100 cancer genes ($G_{T}$), an odds ratio (OR) is computed such that OR > 1 indicates that OL mutations are more likely to occur in $G_{T}$ and OR < 1 indicates that neuronal mutations are more likely to occur in $G_{T}$. Formally, OR = (# OL sSNVs in $G_{T}$/# genic OL sSNVs not in $G_{T}$)/(# neuron sSNVs in $G_{T}$/# genic neuron sSNVs not in $G_{T}$). See also Figure S7.

Patterns of somatic mutation in cancer often contain sufficient information to identify the cell type from which a tumor emerged^58^; thus, we explored whether our normal OL sSNV densities resembled those from a large collection of cancer WGS data from the Pan-Cancer Analysis of Whole Genomes (PCAWG) project.^52^ OL sSNVs were positively correlated with somatic mutation densities of all cancer types from PCAWG, whereas neuronal sSNVs were not correlated with any tumor type (Figure 7A). Specifically, for OL mutations, the highest correlations observed corresponded to glioblastoma multiforme (CNS-GBM) for which OPCs are thought to be the cell of origin.^25,26,36,37^

Our snATAC-seq data allowed further cell-type-specific evaluation of cancer sSNV densities. Among all tumor types in PCAWG, GBM sSNV density was best predicted (with a negative coefficient) by OPC-specific snATAC-seq tracks using a regression model, with 47% of variance in GBM sSNV density explained (Figure 7B). The correlation between cancer sSNV density and snATAC-seq accessibility was negative in all cases, consistent with the negative association between OL mutations and snATAC-seq accessibility. This provides additional evidence that OPCs are the cell of origin for GBM tumors and that snATAC-seq is a powerful approach for determining the cell of origin for a tumor.^58^ Expression levels from snRNA-seq were far less effective in explaining cancer mutation density, explaining only 6% of variance in the best case (Figure 7C).

Finally, we tested whether cancer-associated genes were more likely tobe mutated in OLs compared with neurons. For each tumor type, we determined the 100 most-frequently mutated genes and computed an odds ratio (OR) to assess whether mutations in OLs (OR > 1), neurons (OR < 1), or neither cell type (OR = 1) were more likely to occur in the frequently mutated genes. In general, OL sSNVs were biased toward cancer-associated genes (OR near 1.1, Figure 7D) for most cancer types, likely reflecting the overall correlation between OL sSNVs and cancer mutation densities. Because the gene ranking was not controlled for gene length, the mutations in the most frequently mutated genes may be driven by shared background mutation rates (e.g., increased mutation density in closed chromatin^38^) rather than gene-specific effects. Nevertheless, OL sSNVs were clearly biased toward genes mutated in CNS tumors, with the highest ORs observed for oligodendrogliomas (CNS-Oligo, OR = 1.23, p = 5.3 × 10^−6^, Fisher’s exact test) and pilocytic astrocytomas (CNS-PiloAstro, OR = 1.22, p = 2.0 × 10^−5^). Analysis of the top n cancer mutated gene lists for n = 1–500 confirmed that these findings did not depend on our choice of cutoff n = 100 (Figure S7). Altogether, the similarities between OL—especially those acquired at the OPC stage— and cancer mutation patterns point toward the possibility of a contributory relationship to tumorigenesis.

## DISCUSSION

Our integrative analysis of somatic mutations uncovered OL-specific mutational processes during aging compared with neurons; furthermore, similarities between enriched locations of OL mutations and cancer mutations in the genome suggest that OL mutagenic processes may be related to cancer initiation or progression. Our study design provides an opportunity to explore how different cell types sharing the same microenvironment for years—or even decades—can exhibit contrasting mutational features. An additional advantage of our design is that comparison of OLs and neurons using the same single-cell DNA sequencing technology helps to rule out the possibility that differential mutation rates or genomic distributions reflect technical artifacts or biased representation of specific genomic regions.

Somatic mutation burdens increase linearly in both OLs and neurons with age; however, OLs accumulate 81% more sSNVs and 28% fewer indels than neurons. The apparently lower indel rate in OLs may reflect a high rate of indel mutagenesis in neurons compared with other cell types, as reported by previous studies.^9^ Although some of the excess sSNVs in OLs (e.g., those attributed to SBS1) are likely associated with cell division in ancestral OPCs,^59^ it is not clear what biological processes account for the remaining excess sSNV burden in OLs. A recent study highlighted the importance of cell proliferation-independent sources of somatic mutations in normal cells and hypothesized that the interplay between cell-type-specific DNA damage and repair processes may underlie differences in mutation burden between cell types.^9^ Hence, less-efficient DNA repair processes in OLs—rather than additional DNA damage—may be a plausible explanation for the excess OL sSNV burden compared with neurons. Follow-up studies mapping DNA repair sites in OLs vs. neurons might be needed to address this question.^60-62^

Mutational signature analysis was helpful in identifying some factors that contribute to the overall mutational burden and to its accumulation over time. OL mutagenesis was primarily characterized by SBS1, SBS5, and SBS32, whereas neurons exhibited mostly SBS5 and SBS16. SBS1 was prevalent in OLs and nearly absent in neurons, consistent with previous characterizations of SBS1 as a cell-division-dependent mutational clock^49^ but at odds with a recent study that estimated a nearly 10-fold greater SBS1 rate in human neurons.^9^ SBS5 made up the majority of mutations in both OLs and neurons, but accumulated at significantly different rates (14.5 vs. 22.7 sSNVs/year in neurons and OLs, respectively) and in different areas of the genome in the two cell types. One attractive explanation for this is differential repair: SBS5-associated DNA damage may occur throughout the genome but be more efficiently repaired in certain genomic regions in a cell-type-specific manner. However, measurements of SBS5 exposure may reflect incomplete deconvolution of SBS5, as represented in the current COSMIC catalog. For example, because COSMIC signatures were generated primarily by cancer exomes and genomes, the signatures present in post-mitotic cells are likely to be under-represented. In addition, despite the dozens of single cells we sequenced, the total number of mutations is not large enough to confidently identify signatures that are present at low exposures.

Mature OLs capture mutations accumulated in both ancestral OPCs—which continue to proliferate throughout life, though at lower rates than in early development—and terminally differentiated OLs. Our analysis of shared sSNVs in OL pairs suggests that the earliest mutagenic processes in OPCs strongly feature SBS1, but that SBS5-like processes emerge later in life. Elevated SBS1 in the MDA-amplified mixed glia population also point to SBS1 as a feature of OPCs, despite only ~58% of this sorted population being OPCs (this increase in SBS1 is unlikely to be explained by differences in PTA and MDA amplification because SBS1 levels were similar in MDA-amplified OLs and PTA OLs). OPC-specific mutagenesis also suggests a greater role for selective forces during aging in OLs. Although aging non-proliferating neurons cannot be subject to positive selection, and negative selection is likely limited to highly deleterious mutations that induce cell death, mutations seen in OLs but gained in OPC ancestors are subject to both positive and negative selective effects. Thus, the subset of OL somatic mutations acquired at the ancestral OPC stage, which can expand clonally and amplify deleterious effects, are of particular importance. Although the implications of these remain unclear, they may prove pertinent to age-related, cell-type-specific pathologies in the human brain.

OL mutations are more prevalent in transcriptionally inactive and/or inaccessible chromatin. OL mutations also resemble patterns reported in cancer^38^ — including mutational signatures active in CNS tumors^47^ and the distribution of mutations across the genome, particularly for GBM, a tumor type for which OPCs are believed to be the cell of origin—and other proliferative cells,^58^ possibly reflecting the propagation of somatic mutations acquired by proliferative OPC ancestor cells into mature OLs. Associations between OL somatic mutation density and genomic covariates generally were not cell-type- or tissue-specific. Neuronal mutations were characterized by strongly contrasting patterns of enrichment in transcriptionally active, open chromatin preference for genomic covariates measured in brain tissue—particularly excitatory neurons—and did not mirror the genomic distribution of cancer mutations.

### Limitations of the study

Because we lack a protocol to reliably sort OPCs, ~400 shared mutations in three related OL pairs were used to approximate the characteristics of mutations acquired at the OPC stage. Direct sequencing of OPCs will be necessary to quantify the extent of mutagenesis at the OPC stage and to confirm the mutagenic features shared between OLs and OPCs. Despite the large number of single cells we sequenced, the number of somatic mutations detected remains small relative to that of typical cancer sequencing projects, possibly affecting the robustness of mutational signature analysis. The relatively low mutation count also limited our mutation enrichment analyses to large genomic regions (10s–100s of megabases). Future studies with greater numbers of mutations will enhance the resolution of enrichment analyses, e.g., to enable analysis of individual genes.

## STAR★METHODS

### RESOURCE AVAILABILITY

#### Lead contact

Further information and requests for resources and reagents should be directed to and will be fulfilled by the lead contact, Christopher A. Walsh (christopher.walsh@childrens.harvard.edu)

#### Materials availability

This study did not generate new unique reagents.

#### Data and code availability

- De-identified human data (single cell whole genome- and single nucleus ATAC-seq) have been deposited at the NIAGADS DSS, and accession numbers are listed in the key resources table. They are available upon request if access is granted. To request access, contact the NIGADS DSS (https://dss.niagads.org/). Previously generated de-identified human data (single neuron whole genome sequencing, single nucleus RNA-seq and bulk whole genome sequencing) are available at dbGaP, and accession numbers are listed in the key resources table. They are available upon request if access is granted. To request access, contact dbGaP (https://dbgap.ncbi.nlm.nih.gov/aa/).
- All original code has been deposited at Zenodo (https://doi.org/10.5281/zenodo.10784220) and is publicly available as of the date of publication. DOIs are listed in the key resources table.
- Any additional information required to reanalyze the data reported in this paper is available from the lead contact upon request.

### EXPERIMENTAL MODEL AND STUDY PARTICIPANT DETAILS

Post-mortem human tissues for 20 neurotypical decedents were originally obtained from the NIH Neurobiobank at the University of Maryland Brain and Tissue Bank and the Boston University UNITE or VA-BU-CLF Brain Bank according to their approved institutional protocols and following consent from individuals and/or next-of-kin. We preformed secondary, non-human subject research on these de-identified specimens and data with approval from the Boston Children’s Hospital Institutional Review Board under protocol S07-02-0087. Among the 20 decedents, 12 were assigned male at birth and 8 were assigned female at birth (Table S1). Male and female data were analyzed together and sex chromosomes were excluded from analysis. The 20 decedents comprised 3 infants (aged 0-2 years), 3 adolescents (aged 15-20 years), 5 adults (aged 40-60 years) and 9 elderly individuals (aged 65-104 years) at time of death. Specific details for all decedents are available in Table S1.

### METHOD DETAILS

#### Matched bulk DNA samples

Matched germline reference genome sequences for each subject are required for somatic mutation detection by SCAN2. Bulk genomic DNA was extracted using the QIAGEN QIAamp DNA Mini or QIAGEN EZ1 kit and sequenced by either Illumina HiSeq 2000, HiSeq 2500, HiSeq X or NovaSeq 6000 machines to a target mean coverage of 30-45X. Bulk sequencing data for all UMB subjects were previously published; new bulk data was generated only for subjects 301159 and 190106.

#### Nuclear isolation and sorting

Isolation of single nuclei using fluorescence-activated nuclear sorting (FANS) for NEUN and SOX10 was performed using a modified version of a previously described protocol.^66,67^ Briefly, nuclei were prepared by dissecting fresh-frozen human brain tissue previously stored at −80°C, dissolved on ice in chilled nuclear lysis buffer (10mM Tris-HCl, 0.32M Sucrose, 3mM MgAc_2_, 5mM CaCl_2_, 0.1mM EDTA, pH 8, 1mM DTT, 0.1% Triton X-100) using a Dounce homogenizer. Lysates were layered on top of a sucrose cushion buffer (1.8M Sucrose, 3mM MgAc_2_, 10mM Tris-HCl, pH 8, 1mM DTT) and ultra-centrifuged for 1 hour at 30,000rcf. Pellets containing nuclei were resuspended in ice-cold PBS 1X supplemented with 3mM MgCl_2_, then filtered, blocked in PBS 1X supplemented with 3mM MgCl_2_ and 3% Bovine Serum Albumin (blocking solution), and stained with an anti-NEUN antibody (Millipore MAB377) previously used for neuronal nuclei isolation,^4,66^ anti-SOX10 antibody (Novus NBP2-59621R), and DAPI. Other antibodies targeting the OL population were also evaluated, KLK6 (Bioss bs-5870R) and CNP (Bioss bs-1000R). Nuclei were washed once with blocking solution, centrifuged at 500rpm for 5 minutes and resuspended again in blocking solution. Single nuclei were sorted into 96-well plates, with one nucleus per well.

#### Whole-genome amplification and sequencing

Whole-genome amplification was performed using Primary Template-directed Amplification (PTA) (ResolveDNA EA Whole Genome Amplification Kit, BioSkryb) or Multiple-Displacement Amplification (MDA) (REPLI-g Single Cell Kit, QIAGEN) following manufacturer guidelines. Libraries for sequencing were generated using the KAPA HyperPlus kit (Roche) using dual indexes and were sequenced across 5 lanes of Ilumina NovaSeq 6000 (2x150bp), targeting 30x coverage (~100 Gbp) per sample. FASTQs were aligned to hs37d5, a variant of hg19 with decoy sequences, using bwa mem 0.7.17-r1188 and postprocessed with GATK 4.0.3.0 following the GATK Best Practices (Picard MarkDuplicates, indel realignment and base quality score recalibration).

#### 10x Single nucleus RNA-seq

##### Sample processing and sequencing

snRNA-seq was performed using the 10X Genomics Chromium Next GEM Single Cell 3' Reagent Kit v3.1. Fresh frozen human brain tissue from the prefrontal cortex of individuals UMB1465, UMB4638 and UMB4643 was processed to obtain nuclear pellets. Briefly, tissue was dissociated on ice in chilled nuclear lysis buffer (10 mM Tris-HCl, 0.32 M Sucrose, 3 mM MgAc2, 5 mM CaCl2, 0.1 mM EDTA, pH 8, 1 mM DTT, 0.1% Triton X-100) using a Dounce homogenizer. Homogenates were layered on top of a sucrose cushion buffer (1.8 M Sucrose, 3 mM MgAc2, 10 mM Tris-HCl, pH 8, 1 mM DTT) and ultra-centrifuged for 1 hour at 30,000 rcf. Pellets containing nuclei were resuspended in 250 ml ice-cold 1X PBS supplemented with 3 mM MgCl2, 3% Bovine Serum Albumin (BSA) and 0.2 U/μl RNAse inhibitor (Thermo Fisher Scientific ref.10777019), then filtered. After filtering, suspension volume was completed to 1 ml using the same solution, and nuclei were stained with DAPI before sorting to select for intact nuclei. Some of the UMB1465 samples were additionally stained with the following antibodies: two samples with anti-NEUN antibody (Millipore MAB377) for neuron sorting, one sample each for anti-CX43/GJA1 (Novus Biologicals, FAB7737R-1 00UG AF647), anti-SOX9 (Abcam, ab196450 AF488) and anti-GFAP (Millipore, MAB3402 AF647) to enrich for glial cells, and one sample with anti-SOX10 (Novus Biologicals, NBP2-59621 AF647) for oligodendrocyte sorting. 10,000 to 15,000 single nuclei were sorted for each experiment directly in a tube containing the 10X RT mastermix, and immediately processed for gel-bead in emulsion (GEM) generation, barcoding, cDNA amplification and library preparation following manufacturer instructions. Each library preparation was submitted for paired-end single indexing sequencing on Illumina HiSeq X or NovaSeq 6000 targeting ~50,000 read pairs per nucleus.

##### Data analysis

snRNA-seq data were demultiplexed using bcl2fastq. snRNA-seq FASTQ files were then processed using the 10x Genomics cellranger count pipeline (v6.0.0) for gene expression to perform alignment to hg19, barcode counting, UMI counting, and generation of feature-barcode matrices. Cell Ranger filtered count matrices were used for downstream analysis using Seurat 3.0.^63^ For each library, we further filtered for cells with > 200 and < 3000 genes and <5% mitochondrial genes, and genes with <10,000 UMI counts and >3 cells. RNA counts were normalized using the LogNormalize method and the 2000 most highly variable features were identified using the vst method. Data were then scaled by regressing out the percentage of mitochondrial genes. We then performed non-linear dimensional reduction and clustering. To remove doublets from our datasets, we ran DoubletFinder^68^ using optimal parameters as per the paramSweep function. Finally, cell-type identities were assigned to each cluster in the Uniform Manifold Approximation and Projection (UMAP) based on expression of known brain cell-type markers. To compute the Pearson correlation of gene expression between pairs of cell types, row means were computed on the expression matrix for cells belonging to each cell type and correlation was computed on the log_10_-scaled mean expression vectors.

#### Nuclear sorting purity

##### Assessment by snRNA-seq

Sorting purity is critical when performing single-cell whole-genome (scWGS) studies. Seven populations of nuclei from individual UMB1465 (two samples stained for NEUN+ and one sample each stained for DAPI, SOX10+/NEUN−, CX43+/NEUN−, SOX9+/NEUN− and GFAP+/NEUN−; see 10x single nucleus RNA-seq; sample processing and sequencing), representing a wide variety of brain cell types, were integrated to determine cell type from gene expression (Figure S1). The control nuclei stained for DAPI+ (3447 nuclei) obtained from a mix of grey and white matter from the PFC identified all the cell-types anticipated for this region; OLs were present at expected levels for white matter.^12^ Of 3739 NEUN+ sorted nuclei, 99% were neurons (Figure S1B), ~1% (40 out of 3739 nuclei) showed markers of the OL population (PLP1/MBP/MOG+), and 0.1% (3 out of 3739 nuclei) expressed the endothelial marker NOSTRIN+. NEUN sorted nuclei can be broadly classified into 60% excitatory and 40% inhibitory neurons consistent with recent reports of excitatory/inhibitory ratios.^69^ Evaluation of 9227 SOX10+/NEUN− sorted nuclei confirmed 99.9% purity for mature OLs, with the absence of other cell-type markers (Figure S1B). The SOX10+/NEUN− sorted nuclei showed homogenous distribution of classic mature OL-markers such as PLP1, MOG, MALAT1, among others. Although SOX10 is expressed in all stages of OL development, including in OPCs, our strategy consistently recovered mostly mature OLs.

##### Assessment by ddPCR

We further confirmed the purity of our oligodendrocyte sorting strategy by droplet digital PCR (ddPCR). Nuclei preparation for FANS was performed as previously described from fresh-frozen brain tissue from individual UMB1465. 2.2 μL cell lysis buffer (0.2 μL lysis enhancer and 2 μL resuspension buffer, Thermo Fisher 11739010) was added to each well of 96-well plates and kept on ice. A total of 232 DAPI+/SOX10+/NeuN− single nuclei and 44 DAPI+ control single nuclei were sorted directly into each well and kept on ice. 3 empty (no nuclei) wells and 9 wells containing 100 nuclei each were additionally prepared as negative and positive controls, respectively. Plates were centrifuged at 500 × *g* for 1 min. at 4 °C to ensure nuclear placement in buffer, followed by lysis at 75 °C for 10 min. A select group of transcripts, corresponding to genes expressed in OLs (*PLP1*, *MBP*) and OPCs (*CSPG4*, *PDGFRA*) and housekeeping genes (*ACTB* and *GAPDH*), were reverse transcribed to generate cDNA using the CellsDirect cDNA synthesis kit (Thermo Fisher 18080200) and TaqMan probes for the transcripts of interest. 60 μL 1X TaqMan probe mix was prepared with 27 μL nuclease-free (NF) water and the following probes: 2X GAPDH (Thermo Fisher 4448490; Assay ID: Hs02786624_g1; Dye: VIC-MGB), ACTB (Thermo Fisher 4448490; Assay ID: Hs01060665_g1; Dye: VIC-MGB), CSPG4 (Thermo Fisher 4351370; Assay ID: Hs00361541_g1; Dye: FAM-MGB), PDGFR1 (Thermo Fisher 4448490; Assay ID: Hs00998018_m1; Dye: VIC-MGB), MBP (Thermo Fisher 4448490; Assay ID: Hs00921945_m1; Dye: VIC-MGB), PLP1 (Thermo Fisher 4351370; Assay ID: Hs01555268_m1; Dye: FAM-MGB). 7.8 uL CellsDirect master mix containing 5 mL 2X SYBR Green Reaction Mix, 0.5 μL 1X probe mix, 2 μL NF H_2_O and 0.3 μL SuperScript III Platinum *Taq* Mix (Thermo Fisher 11736051) was added to each well of the 96-well plates after cell lysis while the plate was kept on a plate cooler. The plate was centrifuged at 500 × *g* for 1 min. at 4 °C followed by incubation at 50 °C for 1 hour, followed by 95 °C for 2 minutes, followed by 23 cycles of 95 °C for 15 seconds and 60 °C for 4 minutes, and an infinite 4 °C step. For ddPCR droplet generation, the CellsDirect-generated cDNA was diluted 1:10 with nuclease-free H_2_O on ice. 19.5 μL ddPCR master mix containing 10.5 μL 2X ddPCR supermix (Bio-Rad 1863026), 1.05 μL VIC probe, 1.05 μL FAM probe, and 6.9 μL nuclease-free H_2_O was added to each well in a new 96-well plate. FAM probes included PLP1 (OL marker) and CSPG4 (OPC marker), while VIC probes included MBP (OL marker) and PDGFR1 (OPC marker). 1.5 mL 1:10 cDNA was added to each well, mixed, and collected by centrifugation. Droplet generation was then performed per the manufacturer protocol, followed by incubation at 95 °C for 10 minutes, followed by 40 cycles of 94 °C for 30 seconds and 60 °C for 1 minute, increasing 1 °C each cycle, followed by 98 °C for 10 minutes, and an infinite 4 °C step. Plates were then read using a ddPCR plate reader (Bio-Rad 1864003).

A total of 44 DAPI+ and 232 DAPI+/SOX10+/NeuN− nuclei were tested for 2 OL markers (PLP1 and MBP) and 2 OPC markers (CSPG4 and PDGFRA). We classified single cells from each population into 4 mutually exclusive groups: 1) OL+/OPC+ cells that expressed at least one of the tested OL and at least one OPC marker; 2) OL−/OPC− cells that expressed none of the tested OL and OPC markers; 3) OL−/OPC+ cells that expressed none of the tested OL markers and at least one OPC marker; 4) OL+/OPC− cells that expressed none of the tested OPC markers and at least one OL marker. Thus, group 1 likely represents cells that are transitioning from OPCs to mature OLs; group 2 represents cell types other than OLs and OPCs; group 3 represents OPCs, and group 4 represents OLs.

#### 10x Single nucleus ATAC-seq

##### Sample processing and sequencing

Nuclei from 10 individuals (infants UMB1278, UMB5817, UMB 5871; adolescents UMB1465, UMB4638, UMB5559; adults UMB4643, UMB5087; elders, UMB5219, UMB5823) from our aging cohort were obtained from the same brain region as used for single cell whole-genome amplification. Tissue was processed as described in nuclear sorting, and nuclei were re-suspended in diluted nuclei buffer provided by the manufacturer. Nuclei derived from different individuals were processed for transposition separately, before loading to the 10x Chromium Controller for GEM generation, barcoding, and library construction, as per manufacturer instructions. Libraries were submitted for paired-end dual index sequencing on one flow cell of Illumina S2 NovaSeq 6000 (100 cycles) to obtain ~50,000 reads per nucleus.

##### Data analysis

Sequencing data were demultiplexed using bcl2fastq and mkfastq. cellranger-atac count v1.1.0 was run separately on the resulting FASTQ files for each snATAC-seq library (one per individual) with default parameters and the vendor-provided hg19 reference. Results from the individual library analyses (Cell Ranger output files fragments.tsv.gz and singlecell.csv from each library) were then merged by cellranger-atac aggr –normalize-depth. snATAC-seq data were analyzed by Signac v1.1.0^64^ and Seurat v3 following the authors’ instructions. Briefly, the merged Cell Ranger output was imported via Read10X_h5 and CreateChromatinAssay; analyzed by RunTFIDF, FindTopFeatures, RunSVD and RunUMAP with LSI reduction; and integrated with our snRNA-seq to assign cell types via GeneActivity, FindTransferAnchors and TransferData.

#### Single neuron whole genome sequencing data

Sequencing data for 52 PTA-amplified single neurons and matched bulks from 17 previously sequenced individuals were downloaded from dbGaP accession phs001485.v3.p1. For individual UMB1465, two matched bulks were used (1465-cortex_BulkD-NA_WGSb and 1465-heart_BulkDNA_WGSb). An additional 4 neurons and 2 bulks from 2 neurotypical individuals were generated for this study (individuals 190106 and 301159) for a total of 56 neurons from 19 individuals. Neurons were re-analyzed by SCAN2 jointly with OLs as described in somatic mutation calling with SCAN2. For a single additional individual (identifier 5171), no PTA neurons or OLs were generated nor were previously sequenced single-cell data used (see Table S1).

#### Somatic mutation calling with SCAN2

SCAN2 v1.1 (commit ID 79ec476) and the associated R package r-scan2 (commit ID aa3d90e) were used for analysis. First, a cross-sample panel (required for indel calling with SCAN2) was built using all 183 BAMs (56 PTA neurons, 66 PTA OLs, 40 MDA OLs and 21 bulks) across 20 individuals. The run was configured via scan2 config with parameters –analysis makepanel –gatk=sentieon_joint; the GRCh37 human reference genome with decoy hs37d5 (–ref), dbSNP v147 common (–dbsnp) and 1000 Genomes phase 3 SHAPEIT2 phasing panel (–shapeit-refpanel) as described in Luquette et al.^10^; one –bam argument for each of the 183 BAMs; and a metadata file passed to –makepanel-metadata mapping each sample ID to an individual ID and amplification type (PTA, MDA or bulk). The panel was then generated via scan2 makepanel. Next, SCAN2 was run in –analysis=call_mutations mode for each individual separately as follows. First, scan2 config was run with –analysis=call_mutations –gatk=sentieon_joint –abmodel-n-cores=10 –sensitivity-n-cores=10; the same GRCh37 reference, dbSNP and phasing panels used for cross sample panel building; and all MDA, PTA and bulk files from the individual were supplied via either –sc-bam (PTA and MDA single cells) or –bulk-bam (matched bulk). The cross-sample panel created above was supplied via –cross-sample-panel. Notably, the –gatk=sentieon_joint option causes SCAN2 to use sentieon driver from Sentieon, Inc. in place of GATK HaplotypeCaller, which greatly reduces runtime. After configuration, SCAN2 mutation calling was then run via scan2 run. Finally, SCAN2 mutation signature-based rescue was run in two batches, one for PTA neuron- and one for PTA OL calls, using scan2 config –analysis=rescue –rescue-target-fdr=0.01 and one –scan2-object flag for each of the 56 PTA neurons or 66 PTA OLs followed by scan2 rescue. MDA OLs and GFAP+/NEUN− mixed glia were also rescued (each as a separate batch) to create uniform data output, but all indel calls and rescued sSNV calls were discarded since SCAN2 does not support these analyses for MDA-amplified single cells. Importantly, SCAN2 does not use mutation signature-based rescue calls for total mutation burden extrapolation and rescued calls were excluded prior to mutational signature analysis. Rescued calls were only used for enrichment analyses in which the mutation spectrum of permuted mutation sets used to model the null distribution is forced to match the spectrum of called mutations, thus controlling for rescue-related biases.

#### Single-cell quality metrics

##### Bulk-accessible autosomal regions

The fraction of the genome amenable to analysis by short read sequencing data was defined by the mean sequencing depth of the unamplified matched bulk WGS data. In more detail, the GRCh37 reference genome was tiled with non-overlapping 100 bp windows and the mean sequencing depths for each of the 21 matched bulk samples was computed in each window. A window was considered bulk-accessible if all bulk samples had mean depth >= 5 (bulk samples were sequenced to ~30X). The fraction of genome passing the minimum depth cutoff for sSNV calling (6 reads) and indel calling (10 reads) in single cell i was defined as the number of bulk-accessible basepairs passing these thresholds in cell i.

##### Median absolute pairwise difference (MAPD)

MAPD quantifies amplification uniformity: a low value indicates high-quality, uniform amplification. For each single cell, the genome is binned into approximately 50 kb bins using the variable-size method, which aims to create bins of equal numbers of alignable bases, following Baslan et al.^70^. Copy numbers ${CN}_{i}$ are computed in each bin i, also following Baslan et al.^70^, and a single MAPD value per single cell is computed via MAPD = median($|{\text{log}}_{2}{CN}_{i}-{\text{log}}_{2}{CN}_{i+1}|$).

##### SCAN2 global VAF-based sensitivity

For each single cell, global sensitivity estimates are computed for SCAN2 somatic mutation detection both including and excluding mutations called by SCAN2’s signature-based rescue procedure. The first estimate $S_{V}$, which excludes signature-based rescue calls and is referred to as VAF-based sensitivity, is computed internally by SCAN2. See location-specific sensitivity correction for further discussion. The second estimate $S_{M}$ includes VAF-based and signature-based calls and is given by $S_{M}\;=\;\text{min}\;(1,S_{V}\left(\left(N_{M}+N_{V}\right)/N_{V}\right))$, where $N_{V}$ and $N_{M}$ are the number of VAF-based mutation calls and mutation signature-based rescue calls, respectively.

##### SCAN2 false discovery rates

SCAN2 false positive rates were previously^10^ estimated for PTA single cells, yielding 0.0131 sSNV errors per megabase and 0.00073 indel errors per megabase for combined VAF-based and mutation signature-based calls. The estimated number of false positive calls per PTA single cell was obtained by multiplying the false positive rate by the number of bulk-accessible megabases (defined above) passing the minimum depth cutoff; the false discovery rate per PTA single cell was the number of false positive calls divided by the total number of VAF-based and rescue calls, capped at a maximum value of 100%. The false discovery rate of the total catalog of mutations for each cell type was the sum of estimated false positive counts over all cells divided by the total number of calls.

#### Total mutation burden estimation

SCAN2 provides estimates of the total somatic SNV and indel burden for each single cell (i.e., the estimated total number of mutations per cell after adjusting for sensitivity of mutation calling). These estimates were obtained from each SCAN2 output RDA file by first load()ing the file in R, then running the SCAN2 function mutburden(). For total sSNV burden estimates in MDA-amplified cells (indels are not called in MDA data), it was necessary to estimate the contribution of the MDA artifact signature and remove it as performed previously.^10^ Briefly, for each MDA cell, sSNVs were fit to the set of active COSMIC signatures with the MDA artifact signature added (see mutational signature analysis), all exposures were then scaled by SCAN2’s total burden extrapolation factor (SCAN2’s mutburden() estimate divided by the number of called sSNVs), and finally the scaled exposures to all signatures except the MDA artifact signature were summed to produce the corrected total mutation burden.

#### Age-related accumulation models

To estimate mutation accumulation rates with age, a mixed-effects linear model was used. These models were fit separately for sSNVs and indels by the R lme4 package^71^ using lmer(genome.burden ~ age*celltype + (1∣individual)), where celltype was either pta_oligo or pta_neuron, individual was the individual ID and age was the numeric age of individual. For analysis of total mutation burden in PTA cells, genome.burden was the value returned by SCAN2’s mutburden() function. For MDA cells, genome.burden was the corrected burden described in total mutation burden estimation. Finally, for aging-related accumulation of individual COSMIC signatures, genome.burden was the value estimated by least squares fitting to the reduced COSMIC catalog. Outlier single cells, defined as cells with abnormally high total sSNV burden and SBS19 burden (n=4, outlier=HIGH) or near absence of any sSNV or indel calls (n=2, outlier=LOW), were excluded from all models of age-related accumulation since they may represent technical artifacts or amplification failure (Table S2). The (1∣individual) component helps to account for variability within and between individuals. Confidence intervals were estimated by confint. For linear mixed models, statistical tests of significance comparing each coefficient, interaction term and intercept to a null hypothesis of 0 were calculated by the lmerTest R package,^72^ which uses a *t*-test based on the Satterthwaite approximation. Throughout the text, these *t*-tests are referred to as LMM (linear mixed model) *t*-tests. When MDA aging rates were estimated, the additional 40 MDA cells were added to the set of PTA cells for model fitting and celltype was allowed to take on the additional values of mda_oligo or mda_gfap to assign these cells to separate groups.

#### Recurrent somatic mutation filtration

Prior to all analyses except quantification of per-cell mutation burden and discovery of closely related oligodendrocyte pairs, somatic mutation calls were filtered to remove duplicates and clusters of mutations in single cells using the digest_calls.R script distributed with SCAN2. VAF-based SCAN2 calls and mutation signature-based rescue SCAN2 calls were combined for the purposes of defining recurrent calls. Exact duplicate mutations (i.e., the same position and base change or indel) that are limited to a single individual likely represent a clonal mutation. In this scenario, a single representative mutation was retained. If the duplicate mutations were instead observed across multiple individuals, we interpreted this as a likely artifact and therefore discarded all instances of the mutation. Clusters of calls within the same single cell, defined as any mutation call within 50 bp of another mutation, were also removed since they suggest underlying alignment artifacts or structural variants. Duplicate and clustered mutations were determined separately for PTA neurons, PTA OLs, MDA OLs and MDA GFAP+ cells. For MDA OLs only, all SCAN2 mutation calls from the 20 OLs from infant brains were additionally filtered prior to duplicate and cluster filtering. These were removed because the mutation burden of young OLs is too small to sufficiently outnumber MDA technical artifacts.

#### SnpEff annotation

Recurrence-filtered SCAN2 somatic mutation calls were annotated for functional impact via SnpEff version 4.3t using the hg19 database. Reported functional impacts were taken from the first ANN field in the SnpEff annotated VCF.

#### Mutational signature analysis

First, SCAN2’s VAF-based mutation calls were converted to VCF format for each single cell. Importantly, SCAN2’s mutational signature-based rescue calls were excluded to avoid possible bias in signature analysis. VCFs were then converted into SBS96 or ID83 spectra using SigProfilerMatrixGenerator version 1.2.17.^65^ Next, the spectra were divided by SCAN2’s total genome burden scaling factor (a single value which maps the number of observed sSNVs or indels to the genome-wide mutation burden estimate). Finally, indel spectra (context_type=ID83) were additionally corrected for SCAN2-specific sensitivity differences between the ID83 channels as described in Luquette et al.^10^

The set of active COSMIC mutational signatures was determined by de novo signature extraction and mapping to COSMIC signatures as performed by SigProfilerExtractor version 1.1.21^48^ to the scaled (and corrected, for indels) spectra. The spectra for all 56 PTA neurons and 66 PTA OLs were provided to a single run of SigProfilerExtractor with parameters: reference genome=GRCh37, minimum_signatures=1, maximum_signatures=6 and nmf_replicates=100 and either context_type=SBS96 or ID83. The suggested solution by SigProfilerExtractor provides the set of COSMIC signatures in the following path: Suggested_Solution/COSMIC_{SBS96,ID83}_Decomposed_Solution/Signatures/COSMIC_{SBS96,ID83}_Signatures.txt. This procedure detected 5 active SBS signatures (1, 5, 16, 19, 32) and 6 active ID signatures (2, 4, 5, 8, 9, 11).

Signature exposure levels were calculated by fitting the scaled (and corrected, for indels) spectra to the set of active COSMIC signatures via non-negative least-squares. For MDA cells only, the MDA artifact signature (Signature B)^4^ was added to the set of active COSMIC signatures. Fitting was performed by the lsqnonneg function from the R library pracma.

#### Location-specific sensitivity correction

SCAN2 does not detect somatic mutations with uniform sensitivity across the genome for several reasons. Some factors are intrinsic to whole-genome amplification (e.g., changes in sequencing depth and allelic imbalance) while others are intrinsic to SCAN2’s mutation model (e.g., the need for germline heterozygous SNPs near candidate somatic mutations). Since differences in location-specific sensitivity could give the appearance of somatic mutation enrichment, it is necessary to control for differences in detection sensitivity. Below, we describe a process by which heterozygous germline variants can be treated as candidate somatic mutations to provide location-specific estimates of somatic detection sensitivity.

A special property of single-cell sequencing data is that germline heterozygous variants and heterozygous somatic mutations should both be present on all sequencing reads from one of two haplotypes. This is a significant difference from somatic mutation detection in bulk sequencing, in which heterozygous germline variants are present at ~50% variant allele fraction (VAF) in diploid regions and somatic mutations are present at a variety of VAFs. Thus, SCAN2’s VAF-based somatic mutation calling procedure is applicable to heterozygous germline variants so long as: (1) SCAN2 filters that remove mutation candidates with support in matched bulk or present in dbSNP are skipped; (2) the germline variant is left out of SCAN2’s local model of allele balance, which is trained at heterozygous SNP (hSNP) sites; and (3) only the single germline variant under assessment is left out of the allele balance model at a time to ensure minimal impact to the model. SCAN2 now applies this “leave-one-out” procedure to all germline heterozygous variant sites determined from the matched bulk (~2 million hSNPs and heterozygous indels per individual) by default, which provides an opportunity to measure sensitivity as a function of genomic position, i.e., location-specific sensitivity.

For a sufficiently large (i.e., containing enough germline variants to estimate sensitivity to a few decimal places) genomic region R, SCAN2’s somatic mutation detection sensitivity in single cell i, $S_{R,i}$, is given by the fraction of heterozygous germline variants passing the minimum sequencing depth requirements in R that are called by SCAN2 in cell i under the leave-one-out procedure:

$$\;S_{R,i}=\frac{1}{|G|}\sum_{g\in G}I(g\;\text{is called by the leave-one-out procedure}),\;G=\{\text{germline het}.\;\text{variants with sequencing depth}\geq \text{min}.\;\text{req}.\;\text{depth}\},$$

where I is the indicator function. $S_{R.i}$ is calculated separately for somatic SNVs (where G is the set of hSNPs) and indels (where G is the set of heterozygous germline indels). The condition that G contain only germline variants that meet the minimum sequencing depth is imposed to correspond to SCAN2’s permutation tool, in which permuted mutations for cell i are uniformly distributed over the subset of the genome that meets the minimum sequencing depth requirements (6 reads for somatic SNV calling; 10 reads for somatic indel calling).

The same procedure does not apply to mutation signature-based SCAN2 rescue calls because the use of signatures in calling may introduce signature-related sensitivity bias. Therefore, germline variants, which in general do not have the same signature as somatic mutations, are inappropriate controls for determining sensitivity of signature-based rescued calls. Instead, we make the simplifying assumption that the false discovery rate (FDR) among VAF-based SCAN2 calls is similar to the mutation-signature based rescue calls. The validity of this approximation is supported by analyses presented in Luquette et al.^10^ in which the FDR of VAF-based SCAN2 calls was comparable to the FDR of combined VAF-based and rescued SCAN2 calls (Extended Data Figure 2 of Luquette et al.^10^). Under this assumption, the rescue sensitivity $S_{R,i}^{*}$ in region R for cell i can be approximated by scaling the VAF-based sensitivity $S_{R,i}$ by the relative increase of mutation signature-based rescue calls $M_{i}$ compared to VAF-based calls $V_{i}$:

$$S_{R,i}^{*}=\text{min}\left\{1,S_{R,i}\cdot \frac{V_{i}+M_{i}}{V_{i}}\right\}$$

To compute sensitivity for a group G of single cells, the sensitivities of individual cells are weighted by the fraction of mutations contributed to G by cell $i.W_{R,G}^{*}=\sum_{i\in G}\left\{\frac{N_{i}}{\sum_{i\in G}N_{i}}S_{R,i}^{*}\right\}$ where $N_{i}=V_{i}+M_{i}$. When only VAF-based calls are used for enrichment analysis (e.g., COSMIC signature exposure analysis), the calculation proceeds by replacing $N_{i}$ with $V_{i}$ and $S_{R.i}^{*}$ by $S_{R,i}$.

#### Mutation enrichment analysis

##### Defining genomic regions

Regions for enrichment analysis were defined by first constructing a mask for short, paired-end read alignability and then mapping quantitative and non-quantitative covariates onto the alignable subset of the genome. To determine alignable regions, the human reference genome GRCh37 with decoy sequences hs37d5 was divided into non-overlapping windows of 100 bp and the average sequencing depth across all PTA neurons and OLs as output by SCAN2 (file path: path/to/scan2_output/depth_profile/joint_depth_-matrix.tab.gz) was computed for each 100 bp window. A single mask applicable to all samples was created by classifying windows with low average depth (<6 reads averaged across all PTA cells) or excessive average depth (in the top 2.5% of average depth) as unalignable. Next, genomic regions were derived from non-quantitative genomic covariates (genic and intergenic spaces, Figures 1D and S4E; genes, Figure S4F; ChromHMM classes, Figure 5F; and cell-type-specific promoters and enhancers, Figure 5G) and quantitative covariates (GTEx transcription levels, Figure S5B; snRNA-seq transcription levels, snATAC-seq accessibility, RepliSeq replication timing and histone mark levels, Figures 5B and 5D-5G). For non-quantitative covariates, regions R were defined by the union of genomic intervals for each unique covariate state (e.g., all exons or all regions annotated as ChromHMM state 1) and unalignable windows were subtracted from these unions. For quantitative covariates, the genome was first tiled with non-overlapping 1 kb windows (corresponding to 10 100 bp windows from the alignability mask). 1 kb windows containing >2 unalignable windows were discarded. For each remaining 1 kb window i, a single quantitative value $V_{i}$ was derived for each covariate in a covariate-dependent manner (described in detail for each covariate below). The distribution of values $V_{i}$ were then discretized into n = 10 (for enrichment analysis of total mutation burden) or n = 3 quantiles (for enrichment analysis of mutation signatures) and each window i was assigned its quantile rank $Q_{i}$. Finally, a region $R_{Q}$ was defined for each quantile Q=1…n by taking the union of windows with rank Q. Genomic region construction and the following enrichment analyses were always performed using one covariate at a time.

##### Genomic covariates

GENCODE genes version 26 was downloaded from https://ftp.ebi.ac.uk/pub/databases/gencode/Gencode_human/release_26/GRCh37_mapping/gencode.v26lift37.annotation.gtf.gz. The GTF was fed into the GTEx project’s transcript collapse script to create one unified transcript per gene (https://raw.githubusercontent.com/broadinstitute/gtex-pipeline/master/gene_model/collapse_annotation.py), after which only “gene” records (column 3=gene) located on an autosome were retained. This gene model was used for the per-gene enrichment analysis in Figure S4D (due to the small size of individual genes, somatic detection sensitivity correction was not performed). Genic regions, as analyzed in Figure 1D, were then defined as the union of all transcripts in this gene model; intergenic regions were defined as the complement. 15-state ChromHMM annotations were downloaded for epigenome ID E073 (dorsolateral prefrontal cortex) from https://egg2.wustl.edu/roadmap/data/byFileType/chromhmmSegmentations/ChmmModels/coreMarks/jointModel/final/E073_15_coreMarks_mnemonics.bed.gzhttps://egg2.wustl.edu/roadmap/data/byFileType/chromhmmSegmentations/Chmm Models/coreMarks/jointModel/final/E073_15_coreMarks_mnemonics.bed.gz. Active promoter and enhancer elements for specific brain cell types were extracted from Supplementary Table 5 of Nott et al.^54^. Duplicate lines in these tables were removed prior to analysis. Median gene expression levels from 54 tissue types were downloaded from the GTEx project at https://storage.googleapis.com/gtex_analysis_v8/rna_seq_data/GTEx_Analysis_2017-06-05_v8_RNASeQCv1.1.9_gene_median_tpm.gct.gz. For each tissue type, the median transcription level of each gene G was mapped to the genome by applying it over G’s collapsed transcript (defined by GENCODEv26 gene transcripts, see above). That is, each basepair in the genome overlapped by gene G was assigned the transcription level of G (in TPM). Bases not overlapped by any gene were assigned an expression level of 0. When multiple genes overlapped, the basepairs in the overlapping area were assigned maximum expression value among the overlapping genes. The genome was then tiled into 1 kb windows and windows for which <20% of the window was overlapped by a transcript—regardless of expression level—were removed. Finally, each 1 kb window was assigned a single TPM value by averaging the assigned TPM values over the 1,000 basepairs in the window. Cell type-annotated gene-expression matrices for each snRNA-seq library were concatenated column-wise and average expression levels for each gene were calculated for each cell type. Gene names were then matched to the GTEx gene model and transcription levels for each cell type were mapped to the genome as described above for GTEx transcription levels. snATAC-seq transposition events output by cellranger-atac (file: fragments.tsv.gz) were first separated by cell type (see 10x single nucleus ATAC-seq; data analysis) and then converted to BED format. The BED file of fragments for each cell type was then converted to bedGraph format using bedtools genomecov-bga and finally to bigWig format by bedGraphToBigWig. The bigWig signal files were then mapped to the 1 kb genome tiles or quantitative covariates described in Definition of genomic regions by the bigWigAverageOverBed tool. WaveSignal RepliSeq bigWigs were downloaded from http://hgdownload.cse.ucsc.edu/goldenpath/hg19/encodeDCC/wgEncodeUwRepliSeq/wgEncodeUwRepliseq{cell_line}WaeSignalRep1.bigWig for 15 cell_lines (the full set of available cell lines at the time of analysis): BG02ES, BJ, GM06990, GM12801, GM12812, GM12813, GM12878, HUVEC, HeLa-S3, HepG2, IMR90, K562, MCF-7, NHEK and SK-N-SH. The bigWig signal files were then mapped to the 1 kb genome tiles for quantitative covariates described in mutation enrichment analysis: defining genomic regions by bigWigAverageOverBed; quantile values were then reversed so that Q=1 corresponded to the earliest replication timing quantile. bigWig signal files representing ChIP-seq fold-change versus a no-IP control were downloaded for epigenome ID E073 (dorsolateral prefrontal cortex) from https://egg2.wustl.edu/roadmap/data/byFileType/signal/consolidated/macs2signal/foldChange/E073-{histone_mark}.fc.signal.bigwig for 7 histone_mark values H3K27ac, H3K27me3, H3K36me3, H3K4me1, H3K4me3, H3K9ac and H3K9me3. The bigWig signal files were then mapped to the 1 kb genome tiles for quantitative covariates described in mutation enrichment analysis: defining genomic regions by bigWigAverageOverBed.

##### Estimating mutation enrichment

Enrichment analyses following the methodology described in Luquette et al.^10^ were carried out to determine whether somatic mutations or exposures to COSMIC signatures accumulate preferentially in certain areas of the genome. First, to act as a null hypothesis, a set of permuted somatic mutations was generated for each single cell i by randomly shuffling the positions of the mutation calls in cell i over the subset of the genome meeting the minimum sequencing depth requirements for mutation calling. Only VAF-based calls were permuted for COSMIC signature exposure enrichment analysis; VAF-based and mutation signature-based rescue calls were used for total mutation density enrichment analysis. In both cases, only calls that passed recurrence filtration were permuted (see recurrent somatic mutation filtration). Permuted mutation sets were constructed such that both the number and mutation signatures of the provided calls were preserved. 10,000 such iterations of permuted sets were created via scan2 –analysis=permtool –permtool-n-permutations 10000 for each group G of single cells: PTA neurons, PTA OLs, MDA OLs and MDA GFAP+/NEUN− mixed glia.

##### Adjustment for location-specific sensitivity

Adjustments were made to account for differences in mutation detection sensitivity between genomic regions. Briefly, observed mutation counts in each individual region were first adjusted using region-specific sensitivity estimates defined above. Similar adjustments are not applicable to the null hypothesis mutations created by permutation which were uniformly distributed (i.e., represented no difference in calling sensitivity) over the subset of the genome meeting the minimum depth requirements for SCAN2 calling. Instead, to make the sensitivity-adjusted counts comparable to permuted null counts, the sum of sensitivity-adjusted counts across regions was normalized to maintain the original number of observed mutations across regions. Thus, the null hypothesis mutations represent no region-specific sensitivity bias and the observed mutations are adjusted to remove bias to enable a proper comparison.

In more detail, given a region $R_{C}$ defined by genomic covariate C (see mutation enrichment analysis: defining genomic regions) and a group of single cells G, let $N_{R_{C},G}$ be the count of observed mutations in region $R_{C}$ over cells in G:

$$N_{R_{C},G}=\sum_{\text{single cell}\;i\;\text{in}\;G}N_{R_{C},i}$$

The uncorrected enrichment level is given by dividing $N_{R_{C},G}$ by the mean number (over the 10,000 permutation iterations) of null hypothesis, permuted mutations from group G in region $R_{C}$. To adjust the mutation count for the sensitivity of cells in group G in region $R_{C}$, the observed count is divided by the weighted group-wide sensitivity estimate defined in location-specific sensitivity correction:

$$A_{R_{C,G}}=\frac{N_{R_{C},G}}{N_{R_{C},G}^{*}}$$

However, adjusting the counts in this way renders them incomparable to the null hypothesis permuted mutations. To enable this comparison—and thus the calculation of enrichment level, defined as the excess or paucity relative to the null—the sum of adjusted counts must be normalized by setting it equal to the original sum of counts. I.e., given sensitivity-adjusted counts $A_{R_{C},G}$ for all regions $R_{C}$ defined by the single genomic covariate C, the normalized scaling factor $F_{R_{C},G}$ is

$$F_{R_{C},G}=\frac{A_{R_{C},G}}{\sum_{R_{C}\in C}A_{R_{C},G}}$$

The final corrected mutation count is $F_{R_{C},G}\cdot N_{R_{C},G}$ and the enrichment level is given by dividing this quantity by the mean (over the 10,000 permutation iterations) number of null hypothesis, permuted mutations in $R_{C}$ for group G.

##### Mutational signature enrichment analysis

For enrichment analysis of COSMIC mutational signature exposures, the above steps were followed, except: (1) somatic mutations called by SCAN2’s mutation signature-based rescue method were not used (i.e., only VAF-based SCAN2 calls were used) and (2) rather than counting the number of mutations in each region R, the mutations in R were fit to the catalog of active COSMIC SBS or ID signatures (see mutational signature analysis) by non-negative least squares (using lsqnonneg from the pracma R package) and the exposure value for each signature was used in lieu of mutation counts. Furthermore, signature exposure analyses were not corrected for somatic detection sensitivity; instead, the regions used for signature exposure enrichment were made larger (classifying the genome into just 3 quantiles rather than 10 deciles) to reduce the extent of differences in mutation detection sensitivity.

##### Enrichment significance tests

Two-sided tests of enrichment significance for each region R and group of cells G were obtained via a permutation test strategy, described in Luquette et al.^10^ The distribution of enrichment values in region R for group G under the null hypothesis was approximated by computing the 10,000 uncorrected enrichment values for each of the 10,000 permutation iterations. The *P*-value $P_{R,G}$ describing the significance of enrichment (or depletion) of mutations from group G in region R is then the fraction of null enrichment values with more extreme enrichment (or depletion) values than the sensitivity-adjusted enrichment $E_{R,G}$ for observed mutations. To avoid *P*-values of 0, a minimum of *P* = 0.0001 was enforced.


$$P_{R}=\max\left(\frac{1}{10000},\frac{|\left\{i\;:|\log\;E_{R}^{\left(i\right)}|>|\log\;E_{R}|\right\}|}{10,000}\right).$$

#### Analysis of related oligodendrocyte pairs

##### Detection of shared somatic SNVs

Shared somatic SNVs were determined for each pair of single cells within each individual. A shared sSNV was defined as a SCAN2 call present in at least one of the two cells and for which 2 or more mutation supporting reads appear in the other cell. Private mutations were SCAN2 calls that: (1) did not meet the shared mutation criteria and (2) were supported by 0 reads and a total depth of 6 or greater in the paired cell. The remaining mutations were classified as indeterminate. These heuristics identified three pairs of oligodendrocytes with exceptionally high numbers of shared sSNVs: 5559-Oligo-5 and 5559-Oligo-8, PTA-amplified OLs from individual UMB5559; 5657_OL4 and 5657_OL6, PTA-amplified OLs from individual UMB5657; and GliaLC-4-F11 and GliaLC-4-G10, MDA-amplified OLs from individual UMB5657.

##### Time to most recent common ancestor (MRCA)

The mutation counts shown in Figure 3 represent actual mutation calls. However, to estimate time to the most recent common ancestor (MRCA) by comparison to the rate of mutation accumulation with age, it is necessary to extrapolate the calls to total mutation burden. First, to quantify shared versus private misclassifications, we classified germline heterozygous SNPs (hSNPs) using the same criteria applied to somatic mutations. Essentially all germline hSNPs are shared between single cells from the same individual, thus hSNPs identified as private or indeterminate likely represent amplification-related dropout and allow estimation of the rate of erroneous classifications. Let N be the (unknown) number of shared mutations between two cells, f be the fraction of hSNPs classified as either indeterminate or private (which we regard as a misclassification of a truly shared variant), and s be the number of somatic mutations classified as shared. Then, assuming f equally predicts the rate of misclassification among shared somatic mutations, the total number of shared mutations N=s+fN. Solving for N gives the adjustment N=s∕(1−f). The opposite error, a private sSNV classified as shared, should occur rarely since it requires a random artifact to intersect with a true mutation; we thus assumed this rate to be approximately 0. For comparison to the aging trend line, the shared sSNV count was extrapolated to a genome-wide burden by multiplying N by SCAN2’s calls-to-burden scaling factor S (see total mutation burden estimation; note that pair 3, which was MDA-amplified, was adjusted for signature B). The time to MRCA was calculated as T=(NS−I)∕R, where I and R are the intercept and slope of the OL aging linear model (described in *Age-related accumulation models*), respectively. An interval estimate for T was estimated by replacing I and R with all combinations of their 95% confidence interval bounds (determined by confint) and taking the maximum possible interval among these values. This does not produce a statistical confidence interval, but rather provides some insight into how the uncertainty in our trend line parameters might affect T.

##### Comparison to infant mutational spectra

For comparison to infant neuron and OL SBS spectra, a higher confidence set of VAF-based sSNV calls was created to reduce the impact of PTA artifacts on the spectra. Although PTA and SCAN2 are effective at removing technical artifacts, cells from infant subjects have extremely few somatic mutations and are thus the most challenging cells to analyze. The high confidence sSNV set was created by increasing SCAN2’s calling stringency threshold from the default target.fdr=0.01 to 0.001 by rerunning SCAN2’s call.mutations() method with target.fdr=0.001.

#### Cancer mutation density analysis

##### Defining tumor mutation density

PCAWG cancer somatic SNV and indel mutation catalogs were obtained in MAF format from the ICGC portal (https://dcc.icgc.org/releases/PCAWG/consensus_snv_indel). Hypermutated tumors within each tumor type were identified by Tukey’s method and mutations from these tumors were removed. Next, cancer mutations for each sample were mapped to the 100 bp windows used for determining alignable genomic regions (described in mutation enrichment analysis: defining genomic regions) and the count of mutations in each window was then normalized by the total number of mutations in that sample. Finally, a track representing the mutation density for each tumor type was created by summing normalized window counts across samples from the same tumor type and written in bigWig format via rtracklayer’s export.bw function. Because 1 kb windows contain too few mutations for meaningful correlation analyses with our neuron and OL somatic mutations, the per-tumor bigWig signal files with 100 bp resolution were mapped to a non-overlapping 1 Mb genome tiling by bigWigAverageOverBed. Unalignable 1 Mb windows were then removed following the same requirements for the 1 kb tiling windows described in mutation enrichment analysis: defining genomic regions. Total neuron and OL mutation counts were also determined over these 1 Mb windows and calling sensitivity and correction was applied (see location-specific sensitivity correction). Correlations were computed between corrected somatic mutation density in either OLs or neurons and each tumor type (Figure 7A). A similar downscaling of signals from 1 kb resolution to 1 Mb resolution via bigWigAverageOverBed was required for comparing cancer mutation densities to snRNA-seq and snATAC-seq signals (scaled by −1/*x*) as shown in Figures 7B and 7C.

##### Cancer gene odds ratio analysis

Since the sizes of individual genes were too small for enrichment analysis given the size of our catalog of somatic mutations from OLs and neurons, we created a larger genomic region by considering sets of genes. For each PCAWG tumor type T, mutations from non-hypermutated samples were mapped to genes by SnpEff and the count $N_{T,G}$ of mutations in each gene G for tumor type T was tabulated. For each tumor type, the genomic region $R_{T}$ corresponding to the top n most mutated genes was created. In each region $R_{T}$, the rates of OL mutations and neuron mutations impacting the region were compared using the odds ratio

$${OR}_{T}=\frac{\frac{\#\;\text{oligo mutations in}\;R_{T}}{\#\;\text{oligo mutations not in}\;R_{T}}}{\frac{\#\;\text{neuron mutations in}\;R_{T}}{\#\;\text{neuron mutations not in}\;R_{T}}}.$$

Thus, ${OR}_{R}>1$ implies a preference for OL mutations in the genes represented in R and ${OR}_{R}<1$ implies a preference for neuronal mutations in R. Figure 7D presents odds ratios for n=100; Figure S7 presents odds ratios for n=1 to 500 to investigate the effect of varying n.

### QUANTIFICATION AND STATISTICAL ANALYSIS

All of the quantitative and statistical methods, strategies, and analyses are described in the relevant sections of the method details or in the table and figure legends.

## Supplementary Material

Table S1

Table S2

Table S3

4

## Figures and Tables

**Table:** KEY RESOURCES TABLE

REAGENT or RESOURCE	SOURCE	IDENTIFIER
Antibodies		
Mouse monoclonal anti-SOX10 Alexa Fluor 647	Novus Biologicals	Clone SOX10/991; catalog number: NBP2-59621
Mouse monoclonal anti-NeuN Alexa Fluor 488	Millipore	Clone A60; catalog number MAB377; RRID: AB_2149209
Mouse monoclonal anti-Connexin 43/GJA1 Alexa Fluor 647	Novus Biologicals	Clone 578618; catalog number FAB7737R
Rabbit monoclonal anti-SOX9 Alexa Fluor 488	Abcam	Clone EPR14335; catalog number ab196450; RRID: AB_2665383
Mouse monoclonal anti-Glial Fibrillary Acidic Protein Alexa Fluor 647	Millipore	Clone GA5; catalog number MAB3402; RRID: AB_94844
Biological samples		
Post-mortem fresh-frozen human brain prefrontal cortex tissue	NIH Neurobiobank at the University of Maryland Brain and Tissue Bank	UMB1278
Post-mortem fresh-frozen human brain prefrontal cortex tissue	NIH Neurobiobank at the University of Maryland Brain and Tissue Bank	UMB5817
Post-mortem fresh-frozen human brain prefrontal cortex tissue	NIH Neurobiobank at the University of Maryland Brain and Tissue Bank	UMB5871
Post-mortem fresh-frozen human brain prefrontal cortex tissue	NIH Neurobiobank at the University of Maryland Brain and Tissue Bank	UMB4638
Post-mortem fresh-frozen human brain prefrontal cortex tissue	NIH Neurobiobank at the University of Maryland Brain and Tissue Bank	UMB1465
Post-mortem fresh-frozen human brain prefrontal cortex tissue	NIH Neurobiobank at the University of Maryland Brain and Tissue Bank	UMB5559
Post-mortem fresh-frozen human brain prefrontal cortex tissue	NIH Neurobiobank at the University of Maryland Brain and Tissue Bank	UMB4643
Post-mortem fresh-frozen human brain prefrontal cortex tissue	NIH Neurobiobank at the University of Maryland Brain and Tissue Bank	UMB5087
Post-mortem fresh-frozen human brain prefrontal cortex tissue	NIH Neurobiobank at the University of Maryland Brain and Tissue Bank	UMB936
Post-mortem fresh-frozen human brain prefrontal cortex tissue	NIH Neurobiobank at the University of Maryland Brain and Tissue Bank	UMB5451
Post-mortem fresh-frozen human brain prefrontal cortex tissue	NIH Neurobiobank at the University of Maryland Brain and Tissue Bank	UMB5666
Post-mortem fresh-frozen human brain prefrontal cortex tissue	NIH Neurobiobank at the University of Maryland Brain and Tissue Bank	UMB5943
Post-mortem fresh-frozen human brain prefrontal cortex tissue	NIH Neurobiobank at the University of Maryland Brain and Tissue Bank	UMB5572
Post-mortem fresh-frozen human brain prefrontal cortex tissue	NIH Neurobiobank at the University of Maryland Brain and Tissue Bank	UMB5219
Post-mortem fresh-frozen human brain prefrontal cortex tissue	NIH Neurobiobank at the University of Maryland Brain and Tissue Bank	UMB5171
Post-mortem fresh-frozen human brain prefrontal cortex tissue	NIH Neurobiobank at the University of Maryland Brain and Tissue Bank	UMB5657
Post-mortem fresh-frozen human brain prefrontal cortex tissue	NIH Neurobiobank at the University of Maryland Brain and Tissue Bank	UMB5823
Post-mortem fresh-frozen human brain prefrontal cortex tissue	NIH Neurobiobank at the University of Maryland Brain and Tissue Bank	UMB4976
Post-mortem fresh-frozen human brain prefrontal cortex tissue	Boston University UNITE or VA-BU-CLF Brain Bank	301159
Post-mortem fresh-frozen human brain prefrontal cortex tissue	Boston University UNITE or VA-BU-CLF Brain Bank	190106
Critical commercial assays		
ResolveDNA Whole Genome Amplification Kit (Formerly SkrybAmpTM EA WGA Kit)	BioSkryb Genomics	P00001 - 07292022
KAPA HyperPlus Kit	Roche	Kit code KK8514; catalog number 07962428001
PicoGreen binding Quant-iT dsDNA Assay Kit	Thermo Fisher Scientific	Catalog number P7589
SeqCap Adapter Kit	Roche	Catalog number 07141548001
TapeStation HS DS100 Screen Tape	Agilent	Catalog number PN 5067-5584
REPLI-g Single Cell Kit	Qiagen	Catalog number 150345
Truseq DNA PCR-free (350bp insert)	Illumina	
Chromium Next GEM Single Cell 3’ GEM, Library & Gel Bead Kits v3.1	10X Genomics	Catalog numbers PN-1000121 and PN-1000128
Chromium Next GEM Chip G Single Cell Kits	10X Genomics	Catalog numbers PN-1000120 and PN-1000127
Single Index Kit T Set A, 96 rxns	10X Genomics	Catalog number PN-1000213
Chromium Next GEM Single Cell ATAC Library & Gel Bead Kits	10X Genomics	Catalog numbers PN-1000175 and PN-1000176
Chromium Next GEM Chip H Single Cell Kits	10X Genomics	Catalog numbers PN-1000161 and PN-1000162
Chromium i7 Multiplex Kit N, Set A, 96 rxns	10X Genomics	PN-1000084
CellsDirect cDNA synthesis kit	Thermo Fisher Scientific	Catalog number: 18080200
Deposited data		
Single neuron (PTA) and matched bulk whole genome sequencing data	Luquette et al.^10^	[dbGaP]: [phs001485.v3.p1]
Single nucleus RNA-seq data for UMB1465	Bizzotto et al.^8^; This study	[dbGaP]: [phs001485.v2.p1]; [NIAGADS]: [NG00162]
Single nucleus ATAC-seq data for UMB1465	Bizotto et al.^8^	[dbGaP]: [phs001485.v2.p1]
Single oligodendrocyte whole genome sequencing data	This study	[NIAGADS]: [NG00162]
Single neuron and matched bulk whole genome sequencing data for samples 301159 and 190106	This study	[NIAGADS]: [NG00162]
Single nucleus ATAC-seq	This study	[NIAGADS]: [NG00162]
Single nucleus RNA-seq for UMB4638 and UMB4643	This study	[NIAGADS]: [NG00162]
Oligonucleotides		
GAPDH VIC-MGB 2X TaqMan probe	Thermo Fisher Scientific	Assay ID: Hs02786624_g1; catalog number: 4448490
ACTB VIC-MGB 2X TaqMan probe	Thermo Fisher Scientific	Assay ID: Hs01060665_g1; catalog number: 4448490
CSPG4 FAM-MGB 2X TaqMan probe	Thermo Fisher Scientific	Assay ID: Hs00361541_g1; catalog number: 4351370
PDGFR1 VIC-MGB 2X TaqMan probe	Thermo Fisher Scientific	Assay ID: Hs00998018_m1; catalog number: 4448490
MBP VIC-MGB 2X TaqMan probe	Thermo Fisher Scientific	Assay ID: Hs00921945_m1; catalog number: 4448490
PLP1 FAM-MGB 2X TaqMan probe	Thermo Fisher Scientific	Assay ID: Hs01555268_m1; catalog number: 4351370
Software and algorithms		
cellranger 6.0.0	10x Genomics	https://www.10xgenomics.com/support/software/cell-ranger/latest
Seurat 3.9.9.9010	Stuart et al.^63^	https://satijalab.org/seurat/
cellranger-atac 1.1.0	10x Genomics	https://support.10xgenomics.com/single-cell-atac/software/overview/welcome
Signac 1.1.0	Stuart et al.^64^	https://stuartlab.org/signac/
bwa 0.7.17-r1188		https://github.com/lh3/bwa
SCAN2 1.1	Luquette et al.^10^	https://github.com/parklab/SCAN2
GATK 4.0.3.0	Broad Institute	https://gatk.broadinstitute.org
sentieon driver v202112.06	Sentieon, Inc	https://www.sentieon.com
SigProfilerExtractor 1.1.21	Islam et al.^48^	https://github.com/AlexandrovLab/SigProfilerExtractor
SigProfilerMatrixGenerator 1.2.17	Bergstrom et al.^65^	https://github.com/AlexandrovLab/SigProfilerMatrixGenerator
bedGraphToBigWig 2.9	UCSC Genome Browser	http://hgdownload.soe.ucsc.edu/admin/exe/
bigWigAverageOverBed 2	UCSC Genome Browser	http://hgdownload.soe.ucsc.edu/admin/exe/
lme4 1.1_33 (R package)	CRAN	https://github.com/lme4/lme4/
lmerTest 3.1_33 (R package)	CRAN	https://github.com/runehaubo/lmerTestR
Custom scripts for figures and analysis v2.0	This study; Zenodo	https://doi.org/10.5281/zenodo.10784220
SnpEff 4.3t	Cingolani^44^	http://pcingola.github.io/SnpEff/
rtracklayer 1.54.0 (R package)	Bioconductor	https://doi.org/10.18129/B9.bioc.rtracklayer

## Highlights

- Oligodendrocytes accumulate more sSNVs and fewer sIndels than neurons during aging
- Oligodendrocytes and neuronal mutations are enriched in different genomic regions
- Different mutagenic processes shape the oligodendrocytes and neuronal genome
- Oligodendrocyte mutations are distributed similarly to those in brain cancers
