# Supplementary material for: Contrasting somatic mutation patterns in aging human neurons and oligodendrocytes
Source: Cell. Author manuscript; Available in PMC 2024 May 1. (PMC11062076; doi:10.1016/j.cell.2024.02.025)
Supplement: 4 [file NIHMS1982020-supplement-4.pdf]

CellPress
OPEN ACCESS

Supplemental figures

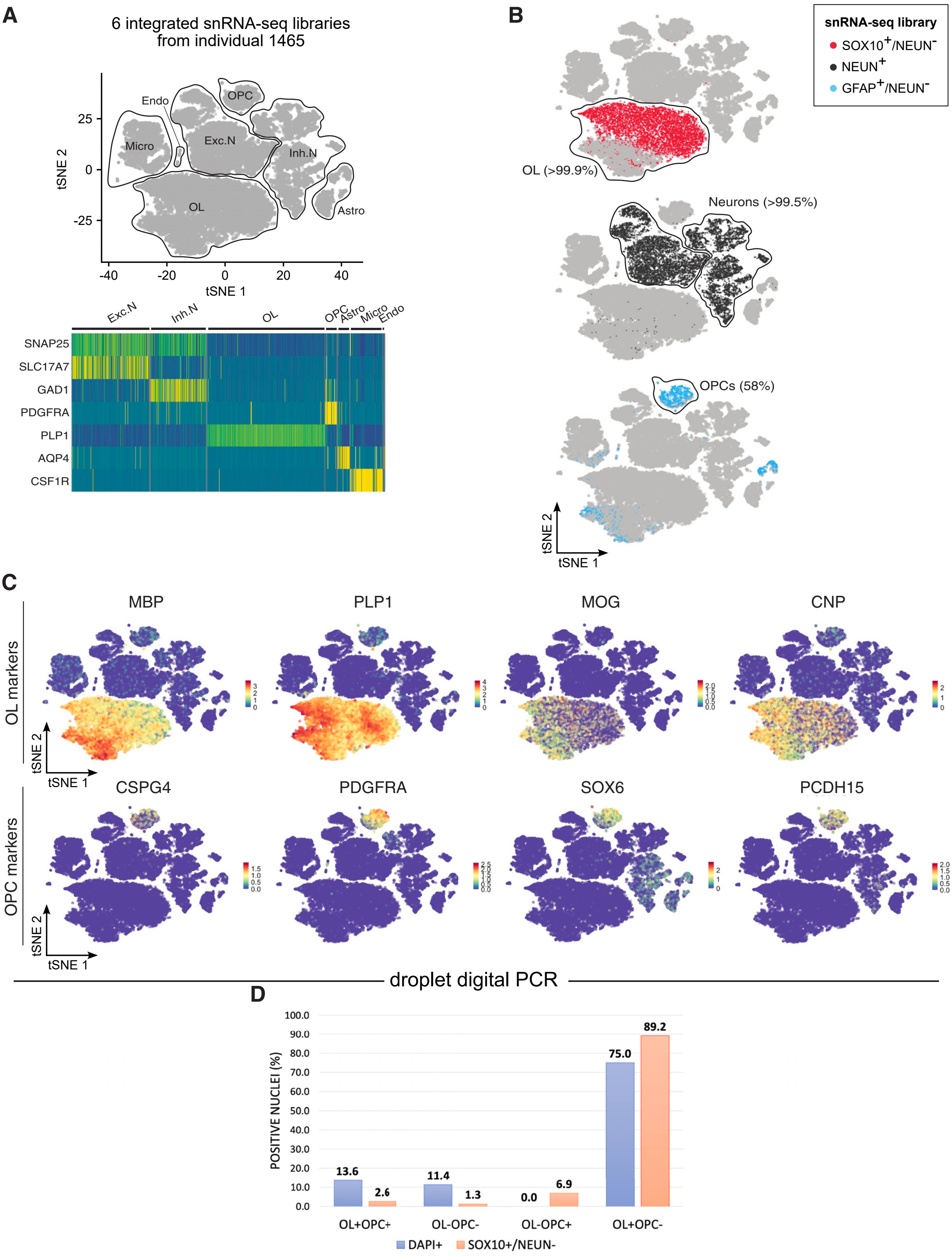

(legend on next page)

Figure S1. Purity of oligodendrocyte sorting assessed by single-nucleus RNA sequencing (snRNA-seq) and droplet digital (dd)PCR, related to Figure 1

(A) Integrated *t*-distributed stochatic neighbor embedding (tSNE) plot of snRNA-seq applied to 6 sorts (DAPI+, NEUN+, SOX10+/NEUN−, GFAP+/NEUN−, CX43+/NEUN−, and SOX9+/NEUN−) of white and gray matter samples from subject UMB1465 (STAR Methods). Major brain cell types were annotated based on the expression of known markers. Representative markers for each cell type are shown in the heatmap.

(B) Using the same tSNE coordinates as in (A), single cells belonging to the SOX10+/NEUN−, NEUN+, and GFAP+/NEUN− sorts are shown. >99.9% of the SOX10+/NEUN− nuclei are found within the oligodendrocyte cluster, while >99.5% of the NEUN+ nuclei clustered as neurons. Nuclei obtained by selecting the GFAP+ population mapped primarily to the OPC cluster (58%), followed by astrocytes (19.7%), oligodendrocytes (18%), microglia (2.6%), endothelial cells (1%), and neurons (0.6%).

(C) Feature plots showing the expression of oligodendrocyte and OPC markers.

(D) Proportion of single nuclei expressing a combination of two oligodendrocyte (PLP1 and MBP) and OPC markers (CSPG4 and PDGFRA) in the SOX10+/NEUN− and DAPI sorts (STAR Methods). OLs, oligodendrocytes; OPCs, oligodendrocyte precursor cells; Exc.Ns, excitatory neurons; Inh.Ns, inhibitory neurons; Astro, astrocytes; Micro, microglia; Endo, endothelial cells.

CellPress
OPEN ACCESS

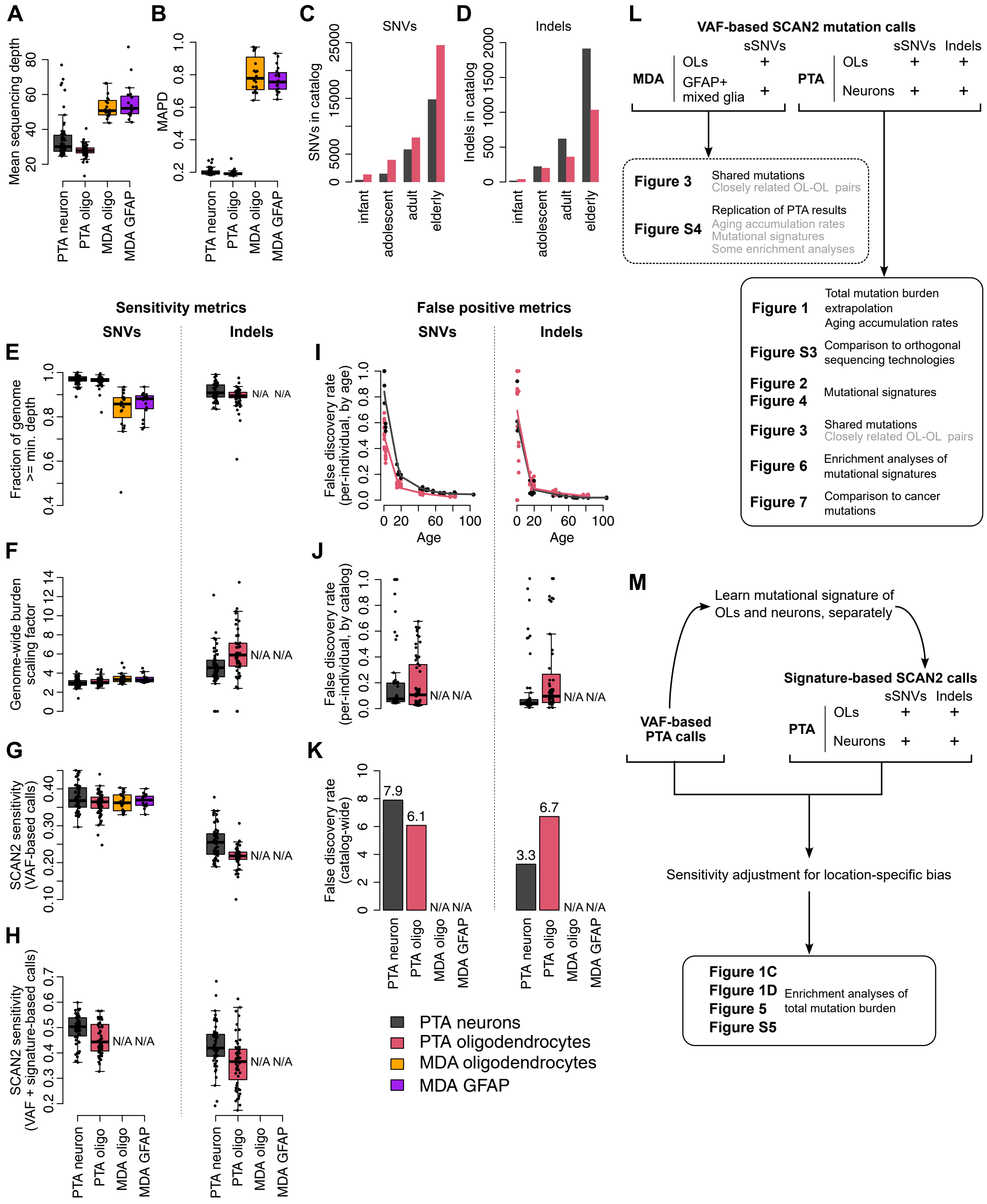

(legend on next page)

Figure S2. Evaluation of SCAN2 somatic mutation detection, related to Figure 1

All boxplots show: median (thick center line), 1st and 3rd quartile (box limits), and furthest outlier not greater than 1.5× the interquartile range from the box limits (whiskers).

(A) Mean sequencing depth per single cell. Each point represents one single cell.

(B) Median absolute pairwise difference (MAPD) per single cell (see STAR Methods), which indicates amplification uniformity along the genome. Lower MAPD values indicate more even amplification.

(C and D) The total numbers of PTA-derived somatic SNVs (C) and indels (D) in our final mutation catalog.

(E) One point per single cell indicating the fraction of the genome passing the minimum sequencing depth requirement for SCAN2 analysis. N/A: SCAN2 does not call somatic indels from MDA-amplified single cells.

(F) One point per single cell indicating the scaling factor S used to extrapolate the number of observed mutations N_V from variant allele fraction (VAF)-based calling (i.e., mutation-signature-based calls are not used for this calculation) to the genome-wide mutation burden. The total burdens, as shown in Figure 1C, are given by SN_V.

(G and H) One point per single cell indicating the sensitivity of VAF-based (G) and mutation-signature-based rescue (H) strategies employed by SCAN2 for somatic mutation detection (STAR Methods). N/A: SCAN2 does not perform signature-based rescue for MDA-amplified single cells.

(I and J) Estimated false discovery rates for each single cell as a function of age (I) and per group (J). The numbers of false positive calls committed per cell were derived from the false positive rate per megabase determined in Luquette et al.[10] (STAR Methods). No such false positive rate estimates were available for MDA.

(K) Estimated false discovery rates across the full catalogs of PTA mutations for each cell type and mutation type.

(L) Mutation types and data types analyzed in this study. VAF-based SCAN2 calling is the first of the two-step mutation discovery process employed by SCAN2.

(M) Mutation-signature-based rescue by SCAN2 was applied to only PTA data. These mutation calls were only used for enrichment analyses with adjustment for signature-related biases.

CellPress
OPEN ACCESS

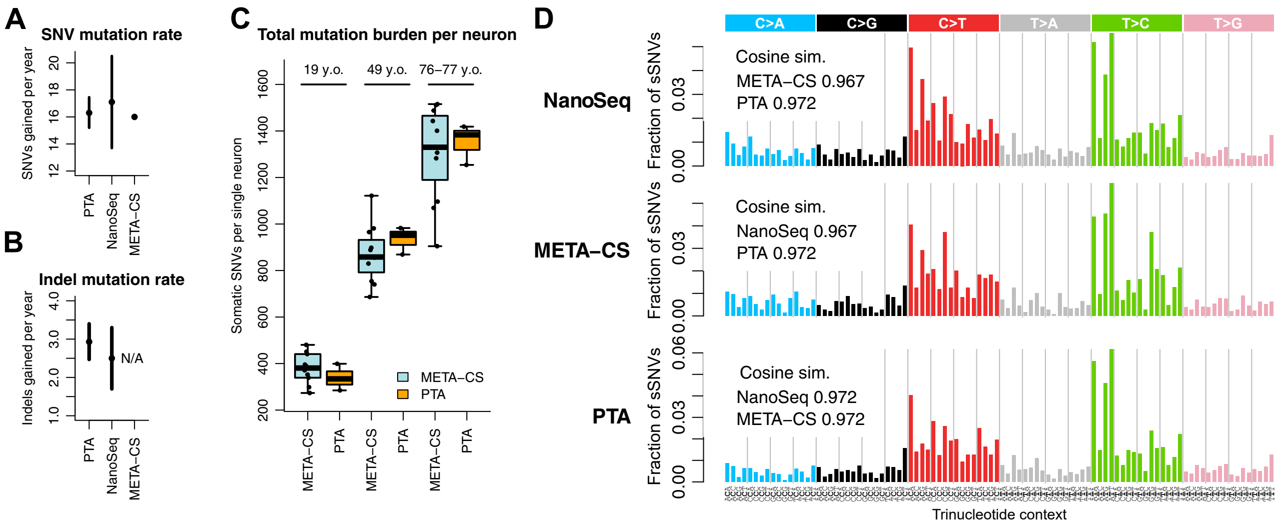

Figure S3. Orthogonal validation of PTA results by two duplex sequencing technologies in human neurons, related to Figure 1

Comparison of two previously published studies of mutations in human neurons (NanoSeq[9] and META-CS[41]) to rates detected in our own neurons support the accuracy of SCAN2 analysis. Both technologies employ duplex sequencing to remove amplification artifacts.

(A and B) Comparison of yearly somatic SNV (A) and indel (B) accumulation rates. Error bars depict the 95% confidence intervals reported previously. Somatic indels were not called in the META-CS study.

(C) Although NanoSeq detects single molecule mutations from bulk cells, META-CS is applied to single cells, allowing comparison of per-cell mutation burden. The three subjects (aged 19, 49, and 76 years) from which neurons were collected in the META-CS study were matched with the closest-aged individuals from our study (UMB5559, 19.8 years; UMB936, 49.2 years; UMB5219, 77 years). For boxplots, the thick center line is the median, box limits are the 1st and 3rd quartile, and whiskers represent the furthest outlier not greater than 1.5× the interquartile range from the box limits.

(D) Comparison of trinucleotide SBS mutation spectra. Cosine sim, cosine similarity.

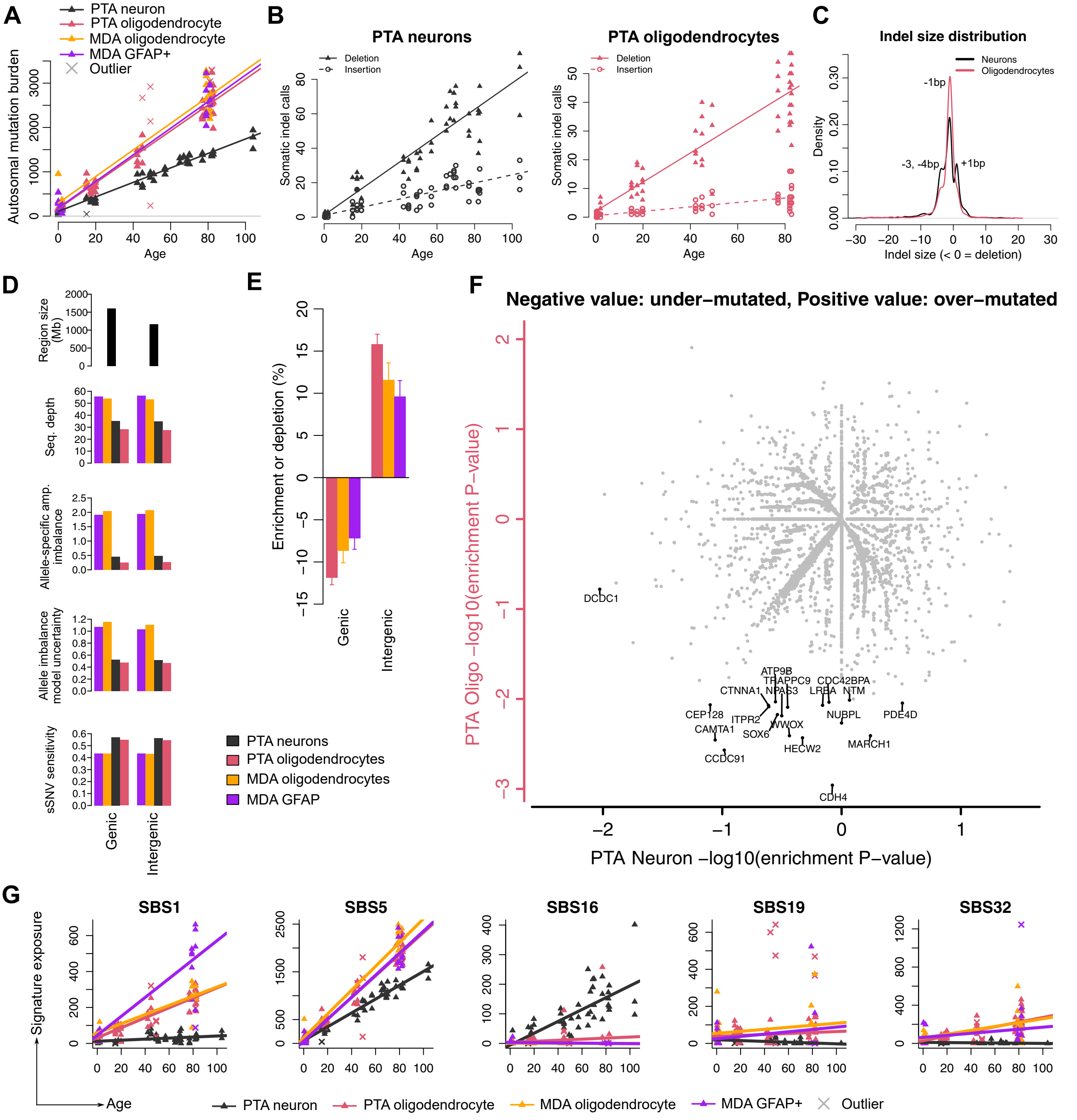

Figure S4. Somatic indel characteristics, comparison of MDA and PTA sSNVs and enrichment analysis of individual genes, related to Figures 1 and 2

(A and B) Aging trend lines for MDA OLs and GFAP+ single cells superimposed on Figure 1C (A) and separate aging trend lines for insertions and deletions (B). Crosses represent outliers. Trend lines are mixed-effects linear regression models from which outliers were excluded.

(C) Distribution of somatic indel sizes. Positive sizes indicate insertions, negative sizes indicate deletions.

(D) Metrics relevant to sensitivity estimation and final somatic mutation detection sensitivity estimates used to correct enrichment analyses. Unless otherwise noted, the plotted values denote the average metric across all PTA neurons (black boxes), PTA oligodendrocytes (red boxes), MDA oligodendrocytes (orange boxes), or MDA GFAP+/NEUN− mixed glia (purple boxes). From top to bottom, the metrics are: size of each genomic region in megabases, which does not change between neurons and OLs; sequencing depth; allele-specific amplification imbalance (lower values indicate more balanced amplification); uncertainty in SCAN2's local allelic imbalance estimates; and mutation-weighted average sensitivity for somatic SNVs and indels. For mutation-weighted sensitivities, rather than take the simple mean, the sensitivity of each single cell is weighted by the fraction of mutations it contributed to the total mutation catalog (STAR Methods).

(legend continued on next page)

(E) Enrichment analyses of genic regions for all PTA OLs compared with MDA cells from elderly subjects (n = 10 OLs and n = 10 GFAP+ mixed glia). The mutation burden of elderly (~80 years of age) OLs is high relative to the rate of technical MDA artifacts, allowing enrichment analysis. Error bars represent bootstrapped 95% CIs (see STAR Methods).

(F) Enrichment analysis of individual genes. Each point represents a single gene. x and y axis values represent significance of enrichment, not enrichment level; significance is signed to represent enrichment (positive significance values) or depletion (negative significance values) of mutations in each gene. Significance values shown are not corrected for multiple hypothesis testing; when corrected, no p values are significant.

(G) Aging trend lines for COSMIC SBS signatures, as estimated by SigProfilerExtractor, for MDA OLs and GFAP+ single cells superimposed on Figure 2C. Crosses represent outliers. Trend lines are linear regressions from which outliers were excluded.

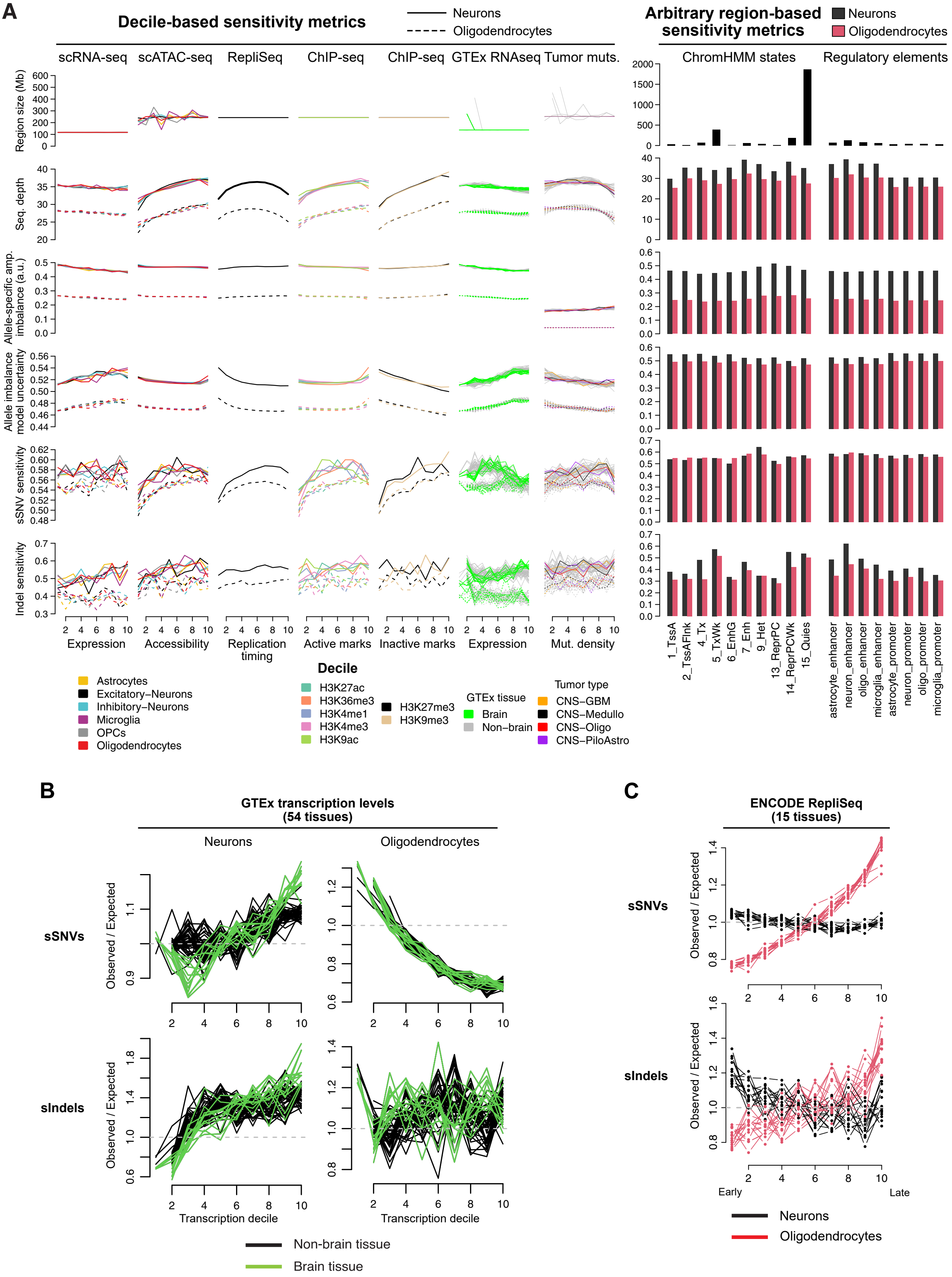

(legend on next page)

CellPress
OPEN ACCESS

Figure S5. Sensitivity metrics for genomic regions analyzed for somatic mutation enrichment and additional enrichment analyses, related to Figure 5

(A) Metrics relevant to sensitivity estimation and final somatic mutation detection sensitivity estimates used to correct enrichment analyses (STAR Methods). Unless otherwise noted, the plotted values denote the average metric across all PTA neurons (solid lines or black boxes) or all PTA oligodendrocytes (dashed lines or red boxes). From top to bottom, the metrics are: size of each genomic region in megabases, which does not change between neurons and OLs; sequencing depth; allele-specific amplification imbalance (lower values indicate more balanced amplification); uncertainty in SCAN2's local allelic imbalance estimates; and mutation-weighted average sensitivity for somatic SNVs and indels. For mutation-weighted sensitivities, rather than take the simple mean across PTA neurons or OLs, the sensitivity of each single cell is weighted by the fraction of mutations it contributed to the total mutation catalog. Notably, tumor mutation densities are calculated over 1 Mb-sized bins, while all other decile-based enrichment analyses are computed over 1 kb-sized bins.

(B) Comparison of somatic mutation density against publicly available bulk RNA-seq transcription levels from GTEx. Each line represents one of 54 tissues in GTEx; green lines, brain tissues; black lines, all other tissue types.

(C) Comparison of somatic mutation burden with 15 ENCODE cell lines for which RepliSeq data are publicly available. The averages of these lines (for neurons and OLs separately) are presented in Figure 5E.

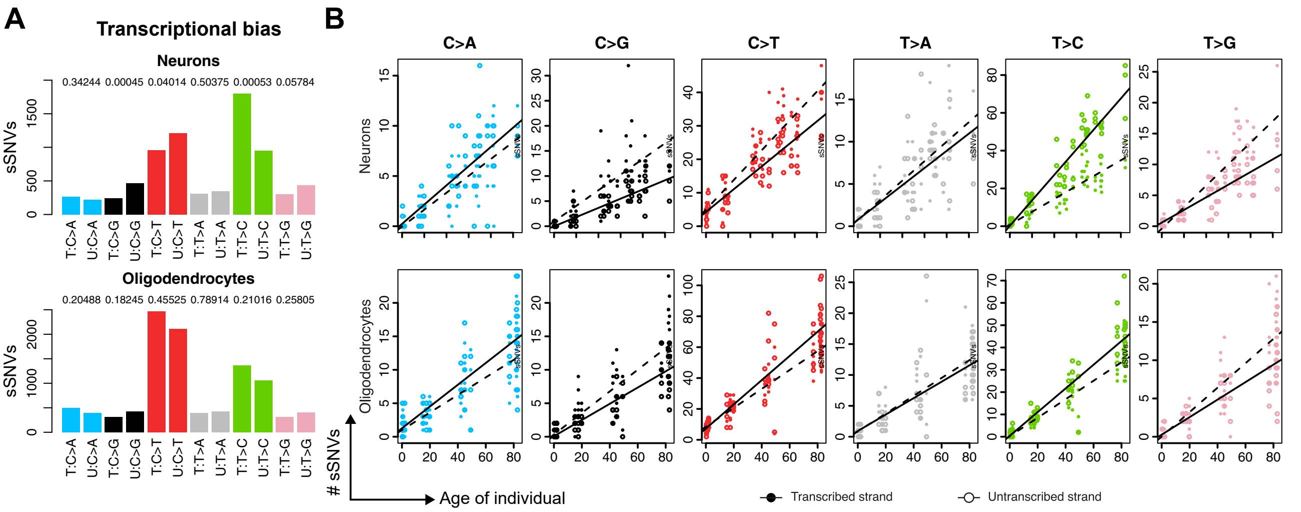

Figure S6. Transcribed-strand bias of somatic SNVs, related to Figure 6

(A) Aggregate transcribed strand bias across all neurons (top) and OLs (bottom). Transcribed-strand status was determined from SigProfilerExtractor's SBS384 output. p values above each pair of bars: for each of the six possible single-base substitutions, Wilcoxon rank-sum test between sample-specific counts of transcribed and untranscribed mutations.

(B) Transcribed (filled circles) and untranscribed (open circles) strand mutation counts plotted separately against age for each single neuron (top) and OL (bottom). Trend lines are linear regression models from which outliers were excluded.

CellPress
OPEN ACCESS

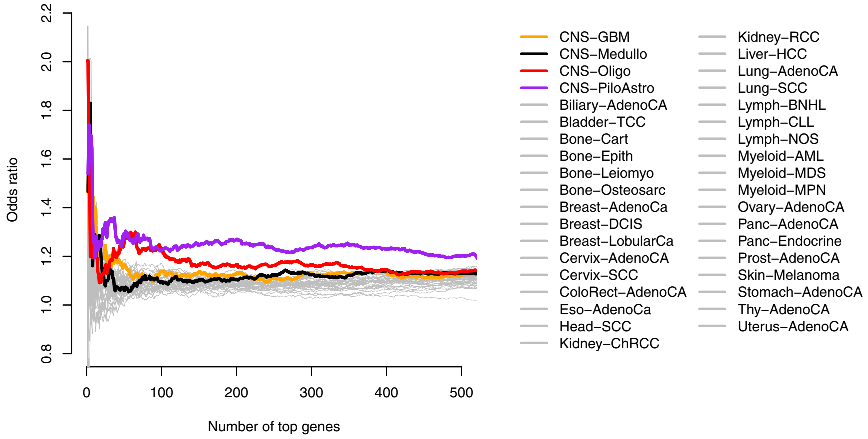

Figure S7. Oligodendrocyte sSNVs remain frequently enriched in brain cancer genes using a wide range of n cutoffs, related to Figure 7

The number n of most frequently mutated cancer genes in the analysis presented in Figure 7D is varied from 1 to 500 to show that choice of n does not alter our conclusions. The analysis in Figure 7D corresponds to n = 100 (x axis value). Each line corresponds to one tumor type; CNS tumors are colored: CNS-GBM, orange; CNS-Medullo, black; CNS-Oligo, red; CNS-PiloAstro, purple.
